# The association between ultra-processed food and common pregnancy adverse outcomes: a dose-response systematic review and meta-analysis

**DOI:** 10.1186/s12884-024-06489-w

**Published:** 2024-05-15

**Authors:** Sepide Talebi, Sanaz Mehrabani, Seyed Mojtaba Ghoreishy, Alexei Wong, Aliasghar Moghaddam, Peyman Rahimi Feyli, Parsa Amirian, Mahsa Zarpoosh, Mohammad Ali Hojjati Kermani, Sajjad Moradi

**Affiliations:** 1 Students’ Scientific Research Center (SSRC), Tehran University of Medical Sciences, Tehran, Iran; 2 Department of Clinical Nutrition, School of Nutritional Sciences and Dietetics, Tehran University of Medical Sciences, Tehran, Iran; 3 Food Security Research Center, Isfahan University of Medical Sciences, Isfahan, Iran; 4 Department of Nutrition, School of Public Health, Iran University of Medical Sciences, Tehran, Iran; 5 Student research committee, School of Public Health, Iran University of Medical Sciences, Tehran, Iran; 6 Department of Health and Human Performance, Marymount University, Arlington, VA USA; 7 Department of Clinical Sciences, Faculty of Veterinary Medicine, Razi University, Kermanshah, Iran; 8 General Practitioner, Kermanshah University of Medical Sciences (KUMS), Kermanshah, Iran; 9 Clinical Tuberculosis and Epidemiology Research Center, National Research Institute of Tuberculosis and Lung Diseases (NRITLD), Masih Daneshvari Hospital, Shahid Beheshti University of Medical Sciences, Tehran, Iran; 10 Department of Nutrition and Food Sciences, Research Center for Evidence-Based Health Management, Maragheh University of Medical Sciences, Maragheh, Iran

**Keywords:** Ultra-processed foods, Pregnancy, Gestational diabetes mellitus, Preeclampsia, Preterm birth

## Abstract

**Objectives:**

Given the increasing incidence of negative outcomes during pregnancy, our research team conducted a dose-response systematic review and meta-analysis to investigate the relationship between ultra-processed foods (UPFs) consumption and common adverse pregnancy outcomes including gestational diabetes mellitus (GDM), preeclampsia (PE), preterm birth (PTB), low birth weight (LBW), and small for gestational age (SGA) infants. UPFs are described as formulations of food substances often modified by chemical processes and then assembled into ready-to-consume hyper-palatable food and drink products using flavors, colors, emulsifiers, and other cosmetic additives. Examples include savory snacks, reconstituted meat products, frozen meals that have already been made, and soft drinks.

**Methods:**

A comprehensive search was performed using the Scopus, PubMed, and Web of Science databases up to December 2023. We pooled relative risk (RR) and 95% confidence intervals (CI) using a random-effects model.

**Results:**

Our analysis (encompassing 54 studies with 552,686 individuals) revealed a significant association between UPFs intake and increased risks of GDM (RR = 1.19; 95% CI: 1.10, 1.27; I^2^ = 77.5%; *p* < 0.001; studies = 44; number of participants = 180,824), PE (RR = 1.28; 95% CI: 1.03, 1.59; I^2^ = 80.0%; *p* = 0.025; studies = 12; number of participants = 54,955), while no significant relationships were found for PTB, LBW and SGA infants. Importantly, a 100 g increment in UPFs intake was related to a 27% increase in GDM risk (RR = 1.27; 95% CI: 1.07, 1.51; I^2^ = 81.0%; *p* = 0.007; studies = 9; number of participants = 39,812). The non-linear dose-response analysis further indicated a positive, non-linear relationship between UPFs intake and GDM risk P_nonlinearity_ = 0.034, P_dose-response_ = 0.034), although no such relationship was observed for PE (P_nonlinearity_ = 0.696, P_dose-response_ = 0.812).

**Conclusion:**

In summary, both prior to and during pregnancy, chronic and excessive intake of UPFs is associated with an increased risk of GDM and PE. However, further observational studies, particularly among diverse ethnic groups with precise UPFs consumption measurement tools, are imperative for a more comprehensive understanding.

**Supplementary Information:**

The online version contains supplementary material available at 10.1186/s12884-024-06489-w.

## Introduction

The Centers for Disease Control and Prevention’s (CDC’s) 2022 National Center for Health Statistics report alarmingly indicates a persistent rise in pregnancy-related mortality in the US across three decades, highlighting significant disparities in “race” and maternal age [1]. This trend underscores the pivotal role of addressing common pregnancy adverse outcomes as a critical component of maternal morbidity and mortality prevention strategies [2].

Promoting healthy dietary habits during pregnancy is imperative to meet the increased physiological needs of expectant mothers. The phenomenon of “nutritional transition”, characterized by a shift towards high-calorie, low-micronutrient foods, culminates in malnutrition and obesity [3]. The significance of maternal nutrition in prenatal care is heavily emphasized by researchers as a preventive measure against adverse pregnancy outcomes [4]. The consumption of diets rich in refined carbohydrates, fats, and sweets is linked to an increased risk of gestational diabetes mellitus (GDM) and preterm birth (PTB) [5]. Moreover, such dietary patterns adversely affect women’s health by exacerbating hypertensive disorders and contributing to conditions like preeclampsia (PE), low birth weight (LBW), and small-for-gestational-age (SGA) infants [6]. Recognizing the detrimental impact of these unhealthy dietary patterns, it becomes crucial to consider the role of food processing in the maternal diet.

The NOVA classification, a framework for grouping edible substances, categorizes foods into four groups based on the extent and purpose of food processing applied, ranging from unprocessed or minimally processed foods to ultra-processed foods (UPFs) [7, 8]. UPFs are characterized by their high content of additives such as preservatives, artificial flavors, colors, and sweeteners, and are typically devoid of whole or minimally processed ingredients [9]. The consumption of UPFs has been associated with higher risks of obesity, hypertension, cancer, and other chronic diseases [8, 10–12]. These foods are implicated in disrupting insulin signaling, promoting excessive energy intake, weight gain, and increased urinary concentrations of phthalate metabolites, which act as endocrine disruptors [13, 14]. In the context of adverse pregnancy outcomes, recent meta-analytic work highlighted a heightened risk of GDM (odds ratio (OR): 1.48; 95% confidence interval (CI): 1.17, 1.87) and PE (OR: 1.28; 95% CI: 1.15, 1.42) among high UPFs consumers, with no significant associations observed in LBW, PTB, and Large for Gestational Age (LGA) [15]. However, the previous meta-analysis did not encompass a comprehensive set of extant studies for each adverse outcome (as evidenced by the inclusion of only 10 studies for GDM in contrast to the 44 studies incorporated in our current investigation), thereby underscoring the challenge posed by the unutilized data in previous analyses. Additionally, recent studies of relevance have emerged [16–18] and the preceding meta-analytic work did not include a dose-response analysis [15]. The integration of dose-response analysis offers benefits such as facilitating the formulation of public health directives, augmenting precision, and quantifying the dose-response relationship. Consequently, we decided to conduct an updated dose-response systematic review and meta-analysis to rigorously evaluate the association between UPFs consumption and common adverse pregnancy outcomes, including GDM, SGA, LBW, PTB, and PE.

## Methods

This systematic review and meta-analysis was conducted according to the guidelines specified in the 2020 Preferred Reporting Items for Systematic Reviews and Meta-Analyses (PRISMA) [19]. The study protocol was registered with the International Prospective Register of Systematic Reviews Database (PROSPERO) under the registration number CRD42023486135.

### Literature search and selection

A systematic literature search was done employing PubMed/MEDLINE, ISI Web of Science and Scopus, with no date restrictions, up to December 6, 2023. The search keywords and strategy are reported in Supplementary Table 1. Data from grey literature sources such as notes, conference abstracts, reviews, case reports, letters, short surveys, and reports were obtained from a manual search of references mentioned in original research articles published in one of these databases. To augment the breadth of research identified, references within reviews and pertinent studies that met eligibility criteria were further subjected to manual examination.

### Inclusion and exclusion criteria

Inclusion criteria were defined as follows: a) observational studies (cohort, case-control, or cross-sectional,) in adult subjects (≥18 years) reporting data on the association between UPFs intake and the risk of adverse pregnancy outcomes (including GDM PE, PTB, LBW, and SGA infants), and reporting effect estimates in the form of hazard ratio (HR), relative risk (RR), or odds ratios (OR), each with at least 95% confidence interval (95% CI). Exclusion criteria included: a) studies done in children and adolescents (< 18 years), b) studies without sufficient data (for instance, those failing to report effect sizes or 95% CIs, instead reporting beta coefficients), and c) those with no relevant exposure. Study titles and abstracts, as well as full-text reviews from database searches meeting the inclusion criteria, were assessed by two reviewers (ST and SM). Any disagreements regarding study inclusion/exclusion criteria were resolved by consensus following discussion. The PICOS tool for each article was reported in Supplementary Table 2.

### Data extraction

Two investigators (FJ and SM) extracted the following data, based on a standardized extraction form, from the studies that met the inclusion criteria: a) first author’s name, year of publication, and country of origin; b) study characteristics (design, follow-up period, and source of data on health status); c) participant characteristics (number of participants/cases, age and sex); d) methods of evaluating UPFs intake; e) the risk of adverse pregnancy outcomes; f) main study results (outcomes), and g) covariates utilized for adjustments in multivariate analyses. Any disagreement regarding data extraction characteristics was decided by consensus following the discussion.

### Quality assessment

Applying the Newcastle-Ottawa Scale (NOS) [20], two investigators assessed the quality of each shortlisted study. The NOS was specifically chosen due to its comprehensive framework designed to evaluate the quality of non-randomized studies. This scale excels in its design, content, and user-friendliness, making it particularly suitable for integrating quality assessments into the interpretation of meta-analytic results. The NOS scale for systematic reviews or meta-analyses, allocating up to 9 points across three domains: study group selection (four points), study group comparability (two points), and exposure and outcomes ascertainment for case-control or cohort studies (three points). Studies scoring 7–9 are deemed high quality/low risk of bias, whereas a score of 0–3 indicates a high risk of bias. Table 1 shows the consensus from this assessment.

**Table 1:** Dietary ultra-processed food and the risk of common pregnancy adverse outcomes

	Highest vs. lowest category meta-analysis						Dose-response meta-analysis							Quality of evidence
	Studies, *n*	Participant/case	RR (95% CI)	*P* value	*I*^*2*^, %	*P*_heterogeneity_	Dose, unit	Studies, *n*	Participant/case	RR (95% CI)	*P* value	*I*^*2*^, %	*P*_heterogeneity_	
GDM	44	180,824/9529	1.19 (1.10, 1.27)	< 0.001	77.2	< 0.001	100 g/day	9	39,812/2326	1.27 (1.07, 1.51)	0.007	81.0	< 0.001	⊕ ⊕ ⊕◯ Medium
PE	12	54,955/4330	1.28 (1.03, 1.59)	0.025	80.0	< 0.001	–	–		–	–	–	–	⊕ ⊕ ⊕◯ Medium
Preterm Birth	8	138,597/5984	1.06 (0.97, 1.17)	0.231	34.2	0.155	–	–		–	–	–	–	⊕ ⊕ ⊕◯ Medium
LBW	4	123,314/672	1.01 (0.91, 1.12)	0.905	52.2	0.099	–	–		–	–	–	–	⊕ ⊕ ⊕◯ Medium
SGA	3	7207/78	1.11 (0.81, 1.52)	0.532	66.3	0.051	–	–		–	–	–	–	⊕ ⊕ ⊕◯ Medium

*RR* relative risk, *CI* Confidence Interval, *GDM* Gestational Diabetes Mellitus, *PE* Preeclampsia, *LBW* Low birth weight, *SGA* Small for gestational age

### Statistical analyses and data synthesis

Statistical analyses were performed applying STATA version 14.0 (StataCorp, College Station, TX, USA) and SPSS version 25.0 (IBM, Armonk, NY, USA). The RR and 95% CI were established as overall effect sizes in this work, similar to effect estimates reported in observational articles meeting the inclusion criteria for this meta-analysis [21]. The synthesized effect estimates were reported as pooled RR with 95% CI. Due to anticipated heterogeneity between studies, effect estimates were calculated using the DerSimonian-Laird weighted random-effects model [22]. A pairwise meta-analysis combined the effect size results for the highest and lowest categories of UPFs intake. Heterogeneity among the articles was examined by the Cochran Q and I-squared (I^2^) statistics, with the I^2^ value estimated from [(Q-df)/Q × 100%]; where Q is the χ^2^ value and df the corresponding degrees of freedom. Between-study heterogeneity was considered significant when the Cochran Q statistic was significant (*p* < 0.05) or if I^2^ > 50%; specifically, low, moderate, high, and extreme heterogeneity was defined based on the I^2^ statistics cut-offs of < 25%, 25–50%, 50–75%, and >75%, respectively.

Furthermore, subgroup analyses were conducted to evaluate any potential effects due to the study design (cross-sectional, case-control, or cohort), UPFs classification method (NOVA food classification, Western-type diet pattern, fast-food, or sweets consumption), the study region of origin (North America, South America, Asia, Europe, and Australia), pre-pregnancy body mass index (< 25 kg/m^2^ and ≥ 25 kg/m^2^) [23, 24], age (< 30 years and ≥ 30 years) [24], number of cases (< 100 or ≥ 100), number of participants (< 1000 or ≥ 1000), dietary assessment method (food frequency questionnaires [FFQ], 24 h recall, or food records), dietary assessment period (pre-pregnancy, early pregnancy, mid-pregnancy), and other covariate adjustments. Sensitivity analysis was conducted by omitting each study and evaluating the remaining pooled effect estimates. Publication bias was evaluated by visual inspection of funnel plots, and formal testing using Egger’s regression asymmetry and Begg’s rank correlation tests [25, 26], with outcomes considered as significant at *p* < 0.05.

A dose-response meta-analysis was completed to estimate the RRs per 100 g increment in UPFs intake, based on the method introduced by Greenland and colleagues [27, 28]. For this process, studies needed to report the number of cases (i.e., participants with incidence) and non-cases (i.e., participants without incidence) or person-years (i.e., the number of people in the study and the duration of their participation) as well as the median point of UPFs intake across more than three categories of intake. Ultimately, a one-stage linear mixed-effects meta-analysis was undertaken to model the dose-response associations, estimating and combining study-specific slope lines to obtain an average slope in a single stage. This linear mixed-effects meta-analysis includes studies with two categories of exposures (at least two effect sizes) in the dose-response analysis.

### Quality of evidence

The quality of evidence across articles was ranked employing the Grading of Recommendations Assessment, Development, and Evaluation (GRADE) working group guidelines. The GRADE criteria categorize evidence quality into high, moderate, low, or very low levels [29].

## Results

### Study characteristics

**Fig. 1:**
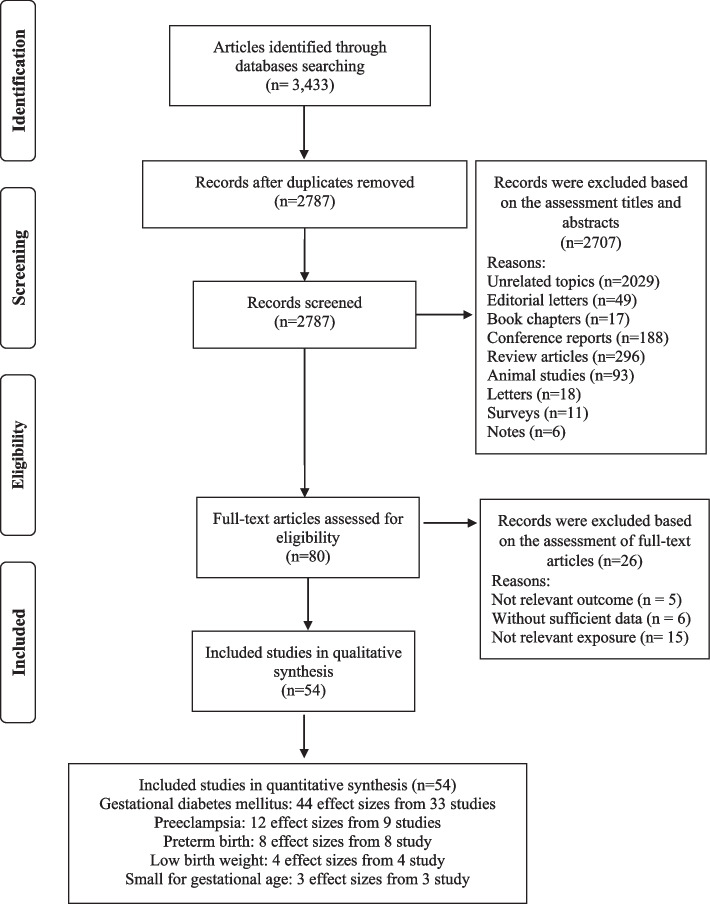
Flow chart of the process of the study selection

Our systematic search and examination of reference lists yielded a total of 3433 records. After omitting duplicates, 2787 articles remained for assessment (Fig. 1). A title and abstract review led to the removal of 2707 articles. Subsequent full-text assessment of the 80 remaining studies resulted in the exclusion of a further 26 articles for the following reasons: five articles reported outcomes not relevant to our research scope, six lacked sufficient data, and 15 did not focus on relevant exposure (Supplemental Table 3). Consequently, 54 studies met our inclusion criteria and were selected in the present meta-analysis [16–18, 30–79].

The selected studies (detailed in Supplemental Table 4) encompass 38 cohort studies [16, 31, 33–37, 39, 40, 42–49, 51, 52, 54, 55, 57–62, 65–67, 69–71, 73–75, 78], 11 case-control studies [17, 18, 30, 32, 34, 50, 63, 66, 68, 72, 77], and five cross-sectional studies [38, 46, 64, 76, 79]. These articles, conducted between 1988 and 2023, originated from different countries including the USA [33, 36, 53, 58, 60, 62, 69, 74, 78], the UK [16], China [43, 49, 51, 71, 73], Brazil [31, 59, 63, 64, 68, 79], Spain [39, 40, 42, 55, 57], Iran [17, 18, 30, 32, 48, 54, 66, 76, 77], Malaysia [75], Palestine [72], Australia [45, 46, 65], Singapore [37, 38], Norway [35, 44, 47], Japan [41, 67], Czech Republic [34], Iceland [70] and Denmark [61]. The study-specific, maximally adjusted RR was reported for 552,686 individuals across the included articles and was pooled for meta-analysis to assess the association between UPFs and the risk GDM [16, 32–34, 36, 38–41, 43, 48–51, 53–56, 59, 60, 64–66, 70–75, 77–79], PE [17, 18, 30, 35, 48, 52, 62, 69, 74, 76], PTB [31, 37, 44–46, 48, 52, 58, 61, 67], LBW [45, 63, 67] and SGA infants [46, 67, 68]. The Newcastle-Ottawa grade (used for quality assessment) categorized 27 studies as high quality [17, 33, 35–37, 39–45, 47, 51, 53, 55, 57, 58, 60, 62, 65, 67, 69, 74, 75, 78] and 27 as medium quality [16, 18, 30–32, 34, 38, 46, 48–50, 52, 54, 56, 59, 61, 63, 64, 66, 68, 70–73, 76, 77, 79]. Moreover, the outcomes revealed that the level of agreement between investigators for data collection as well as for quality assessment was appropriate (Kappa = 0.897).

### Ultra-processed food and common adverse pregnancy outcomes

Our results suggested a significant relationship between higher UPF intake and an increased risk of GDM (RR = 1.19; 95% CI: 1.10, 1.27; I^2^ = 77.5%; *p* < 0.001; *n* = 44), PE (RR = 1.28; 95% CI: 1.03, 1.59; I^2^ = 80.0%; *p* = 0.025; *n* = 12), but not PTB (RR = 1.06; 95% CI: 0.97, 1.17; I^2^ = 34.2%; *p* = 0.231; *n* = 8), LBW (RR = 1.01; 95% CI: 0.91, 1.12; I^2^ = 52.2%; *p* = 0.905; n = 4) and SGA infants (RR = 1.11; 95% CI: 0.81, 1.52; I^2^ = 66.3%; *p* = 0.532; *n* = 3), (Refer to Table 1, Supplementary Fig. 1).

In the context of GDM, subgroup analysis showed that a greater UPFs intake was significantly associated with an enhanced risk in cohort studies (vs. cross-sectional) (RR = 1.18; 95% CI: 1.09, 1.27; I^2^ = 79.3%; *p* < 0.001; *n* = 31) and case-control studies (RR = 2.06; 95% CI: 1.31, 3.35; I^2^ = 77.7%; *p* = 0.002; *n* = 10), particularly in studies assessed western dietary pattern (RR = 1.34; 95% CI: 1.01, 1.76; I^2^ = 43.0%; *p* = 0.040; *n* = 7) or fast-foods (RR = 1.32; 95% CI: 1.15, 1.51; I^2^ = 79.3%; *p* < 0.001; *n* = 22), (vs. NOVA classification or sweets consumption), in North America (vs. Europe, South America, Asia and Australia) (RR = 1.43; 95% CI: 1.27, 1.53; I^2^ = 45.4%; *p* < 0.001; n = 10), and across studies with > 100 number of case (RR = 1.38; 95% CI: 1.21, 1.58; I^2^ = 74.8%; p < 0.001; *n* = 12)(vs. < 100 number of case), in studies with > 1000 number of participants (RR = 1.33; 95% CI: 1.15, 1.54; I^2^ = 76.9%; p < 0.001; *n* = 21)(vs. < 1000 number of participants), in studies used FFQ for dietary assessment (RR = 1.27; 95% CI: 1.14, 1.43; I^2^ = 78.6%; p < 0.001; *n* = 34) (vs. 24 h recall or food record), particularly in studies where the period of dietary assessment was at early pregnancy (RR = 1.26; 95% CI: 1.09, 1.46; I^2^ = 80.5%; *p* = 0.002; *n* = 19) (vs. pre-pregnancy or mid-pregnancy). Moreover, subgroup analysis for covariates adjustment showed that BMI and physical activity may influence the association between UPF intake and the risk of GDM (Table 2).

**Table 2:** Subgroup analyses of ultra-processed food intake and the risk of gestational diabetes mellitus (Highest vs. lowest category meta-analysis)

Sub-groups	Number of effect sizes	Relative Risk (95%CI), *P*_value_	I^2^ (%), P_heterogeneity_	P _between_
Overall	44	1.19 (1.10, 1.27), < 0.001	77.2, 0.001	
Study design				**0.013**
Cohort	31	1.18 (1.09, 1.27), < 0.001	79.3, < 0.001	
Case-control	10	2.06 (1.31, 3.35), 0.002	77.7, < 0.001	
Cross-Sectional	3	0.94 (0.79, 1.12), 0.503	0.0, 0.850	
Ultra-processed food assessment method				**0.998**
NOVA food classification	3	0.99 (0.74, 1.32), 0.920	0.0, 0.673	
Western diet pattern	7	1.34 (1.01, 1.76), 0.040	43.0, 0.104	
Fast-food	22	1.32 (1.15, 1.51), < 0.001	78.8, < 0.001	
Sweets consumption	12	1.01 (0.92, 1.11), 0.857	74.9, < 0.001	
Region				**0.035**
North America	10	1.43 (1.27, 1.61), < 0.001	45.4, 0.058	
South America	3	0.86 (0.64, 1.17), 0.335	0.0, 0.846	
Europe	11	1.13 (1.00, 1.27), 0.051	76.7, < 0.001	
Asia	19	1.12 (0.96, 1.29), 0.149	67.9, < 0.001	
Australia	1	1.23 (0.76, 1.98), 0.394	–	
Number of Case				**0.007**
< 100	12	0.98 (0.94, 1.02), 0.353	30.7, 0.146	
> 100	32	1.38 (1.21, 1.58), < 0.001	74.8, < 0.001	
Number of participants				**0.215**
< 1000	23	1.01 (0.94, 1.09), 0.751	62.0, < 0.001	
> 1000	21	1.33 (1.15, 1.54), < 0.001	76.9, < 0.001	
Age				**0.367**
< 30	21	1.16 (1.06, 1.28), 0.002	65.6, < 0.001	
≥30	19	1.33 (1.14, 1.54), < 0.001	84.3, < 0.001	
Not report	4	0.83 (0.55, 1.26), 0.377	58.6, 0.064	
Pre-pregnancy BMI				**0.021**
≤25	21	1.38 (1.18, 1.60), < 0.001	76.2, < 0.001	
> 25	10	1.53 (1.12, 2.08), 0.07	74.6, < 0.001	
Not report	13	0.99 (0.94, 1.05), 0.808	55.6, 0.008	
Dietary assessment method				**0.229**
FFQ	34	1.27 (1.14, 1.43), < 0.001	78.6, < 0.001	
24 h Recall	7	1.52 (0.99, 2.32), 0.056	73.8, 0.001	
food record	3	1.00 (0.96, 1.03), 0.786	25.0, 0.264	
Dietary assessment period				**0.431**
Pre-pregnancy	10	1.25 (0.97, 1.60), 0.088	73.6, < 0.001	
Early pregnancy	19	1.26 (1.09, 1.46), 0.002	80.5, < 0.001	
Mid-pregnancy	9	1.00 (0.93, 1.08), 0.982	49.3, 0.046	
Not reported	6	2.07 (1.21, 3.52), 0.007	85.7, < 0.001	
Adjustments				
Body mass index				**0.903**
Yes	33	1.25 (1.12, 1.40), < 0.001	77.1, < 0.001	
No	11	1.03 (0.92, 1.16), 0.564	75.0, < 0.001	
Smoking status				**0.398**
Yes	21	1.31 (1.13, 1.51), 0.001	76.7, < 0.001	
No	23	1.05 (0.97, 1.13), 0.269	68.4, < 0.001	
Physical activity				**0.961**
Yes	26	1.25 (1.09, 1.43), 0.001	76.0, < 0.001	
No	18	1.06 (0.97, 1.15), 0.186	70.7, < 0.001	
Alcohol intake				**0.886**
Yes	11	1.24 (1.03, 1.48), 0.019	83.7, < 0.001	
No	33	1.14 (1.05, 1.24), 0.001	71.0, < 0.001	
Energy intake				**0.281**
Yes	27	1.15 (1.07, 1.24), < 0.001	78.2, < 0.001	
No	17	1.42 (1.14, 1.78), 0.002	74.2, < 0.001	

^1^Calculated by Random-effects model

*FFQ* Food Frequency Questionnaire, *BMI* Body mass index

For PE, the subgroup analysis also highlighted that greater UPFs intake was significantly associated with an enhanced risk in studies assessed western dietary pattern (RR = 2.51; 95% CI: 1.13, 5.57; I^2^ = 91.1%; *p* = 0.023; *n* = 3) or NOVA classification (RR = 1.22; 95% CI: 1.04, 1.42; I^2^ = 0.0%; *p* = 0.013; n = 3), (vs. sweets consumption), in Asia (vs. Europe or US areas) (RR = 1.65; 95% CI: 1.07, 2.55; I^2^ = 86.1%; *p* < 0.001; *n* = 6), and across studies with > 100 number of case (RR = 1.57; 95% CI: 1.03, 2.40; I^2^ = 93.2%; p < 0.001; *n* = 4)(vs. < 100 number of case), in studies with number of < 1000 participants (RR = 1.65; 95% CI: 1.07, 2.55; I^2^ = 86.1%; p = 0.023; n = 6)(vs. > 1000 number of participants), in participants aged ≥30 years (RR = 1.28; 95% CI: 1.07, 1.54; I^2^ = 50.4%; *p* = 0.089; *n* = 5)(vs. participants aged < 30 years), in participants with pre-pregnancy-BMI > 25 (RR = 1.52; 95% CI: 1.07, 2.15; I^2^ = 84.7%; *p* = 0.021; *n* = 1)(vs. participants with pre-pregnancy-BMI ≤ 25), in studies used FFQ for dietary assessment (RR = 1.38; 95% CI: 1.10, 1.72; I^2^ = 82.6%; *p* = 0.005; *n* = 10) (vs. questions), and particularly in studies where the period of dietary assessment was at mid-pregnancy (RR = 1.23; 95% CI: 1.05, 1.43; I^2^ = 38.8%; *p* = 0.009; *n* = 3) (vs. early pregnancy). Furthermore, subgroup analysis for covariates adjustment showed that BMI and physical activity may influence the association between UPF intake and the risk of PE (Table 3).

**Table 3:** Subgroup analyses of ultra-processed food intake and the risk of Preeclampsia (Highest vs. lowest category meta-analysis)

Sub-groups	Number of effect sizes	Relative Risk (95%CI), *P*_value_	I^2^ (%), P_heterogeneity_	P _between_
Overall	12	1.28 (1.03, 1.59), 0.025	80.8, < 0.001	
Study design				**0.435**
Cohort	7	1.15 (0.90, 1.46), 0.262	73.3, 0.001	
Case-control	4	1.61 (0.93, 2.77), 0.087	90.7, < 0.001	
Cross-Sectional	1	1.38 (0.46, 4.13), 0.565	–	
Ultra-processed food assessment method				**0.103**
NOVA food classification	3	1.22 (1.04, 1.42), 0.013	0.0, 0.957	
Western diet pattern	3	2.51 (1.13, 5.57), 0.023	91.1, < 0.001	
Sweets consumption	6	0.99 (0.81, 1.21), 0.925	59.9, 0.029	
Region				**0.096**
America	3	0.77 (0.46, 1.28), 0.314	10.5, 0.327	
Europe	3	1.14 (0.89, 1.46), 0.296	82.1, 0.004	
Asia	6	1.65 (1.07, 2.55), 0.023	86.1, < 0.001	
Number of Case				**0.297**
≤100	8	1.15 (0.91, 1.45), 0.073	46.1, 0.073	
> 100	4	1.57 (1.03, 2.40), < 0.001	93.2, < 0.001	
Number of participants				**0.142**
< 1000	6	1.65 (1.07, 2.55), 0.023	86.1, < 0.001	
> 1000	6	1.06 (0.84, 1.34), 0.604	68.0, 0.008	
Age				**0.015**
< 30	3	2.39 (0.78, 7.30), 0.128	80.0, 0.007	
≥30	5	1.28 (1.07, 1.54), 0.008	50.4, 0.089	
Not report	4	0.95 (0.72, 1.25), 0.711	70.1, 0.018	
Pre-pregnancy BMI				**0.353**
≤25	1	1.33 (0.50, 3.54), 0.568	–	
> 25	6	1.52 (1.07, 2.15), 0.021	84.7, < 0.001	
Not report	5	1.07 (0.79, 1.45), 0.661	77.8, 0.001	
Dietary assessment method				**0.115**
FFQ	10	1.38 (1.10, 1.72), 0.005	82.6, < 0.001	
Questions	2	0.64 (0.37, 1.11), 0.115	0.0, 0.428	
Dietary assessment period				**0.039**
Pre-pregnancy	0	–	–	
Early pregnancy	7	1.08 (0.84, 1.40), 0.549	67.1, 0.006	
Mid-pregnancy	3	1.23 (1.05, 1.43), 0.009	38.8, 0.195	
Not report	2	3.11 (0.74, 13.00), 0.025	81.6, 0.020	
Adjustments				
Body mass index				**0.712**
Yes	11	1.30 (1.04, 1.63), 0.023	82.5, < 0.001	
No	1	1.02 (0.52, 1.99), 0.954	–	
Smoking status				**0.441**
Yes	4	1.11 (0.88, 1.40), 0.362	74.5, 0.008	
No	8	1.43 (0.97, 2.12), 0.070	83.2, 0.001	
Physical activity				**0.017**
Yes	5	2.08 (1.15, 3.75), 0.015	82.5, < 0.001	
No	7	1.05 (0.89, 1.23), 0.577	59.0, 0.023	
Alcohol intake				
Yes	0	–	–	
No	12	1.28 (1.03, 1.59), 0.025	80.0, < 0.001	
Energy intake				**0.919**
Yes	5	1.26 (0.98, 1.62), 0.710	77.4, 0.001	
No	7	1.27 (0.83, 1.95), 0.277	84.5, < 0.001	

^1^Calculated by Random-effects model

*FFQ* Food Frequency Questionnaire, *BMI* Body mass index

### Linear and non-linear dose-response analysis

**Fig. 2:**
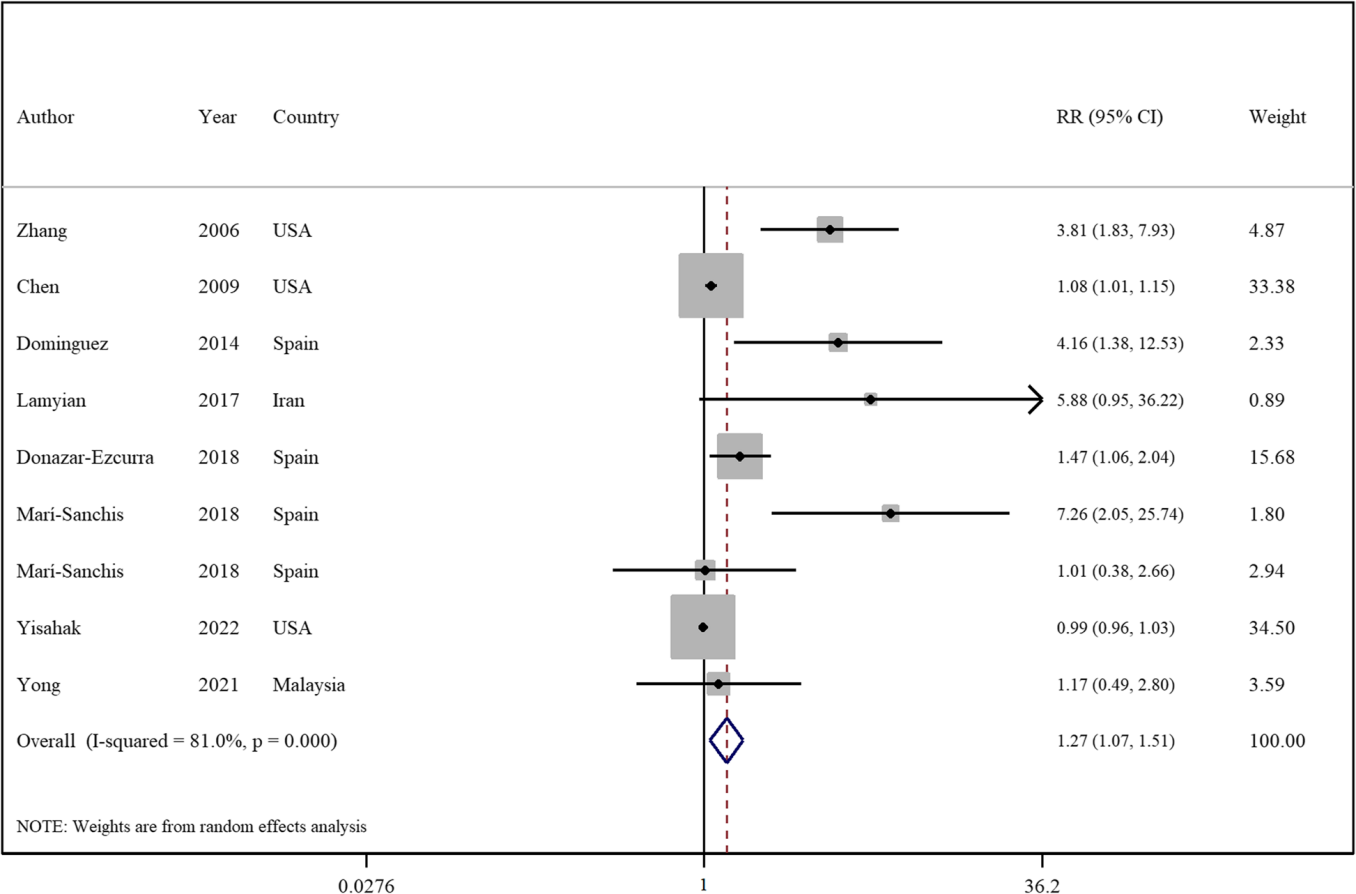
Forest plots showing the linear dose-response meta-analysis of mortality risk for 100 g change in ultra-processed food consumption in daily intake and risk of gestational diabetes mellitus

The linear dose-response analysis (refer to Table 1 and Fig. 2) indicates a 27% increase in GDM risk per 100 g increment in UPF intake RR = 1.27; 95% CI: 1.07, 1.51; I^2^ = 81.0%; *p* = 0.007; *n* = 9). However, the linear dose-response analysis for other outcomes was not undertaken due to the limited number of studies available.

**Fig. 3:**
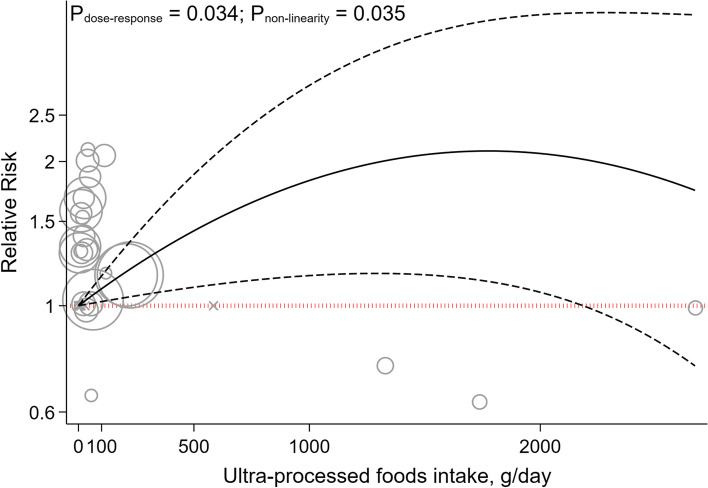
Non-linear dose-response indicated associations between UPF intake and the risk of gestational diabetes mellitus

**Fig. 4:**
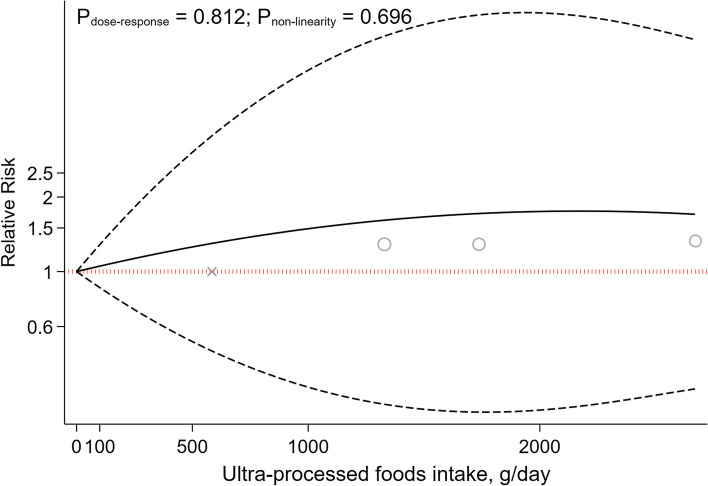
Non-linear dose-response indicated associations between UPF intake and the risk of preeclampsia

The non-linear dose-response analysis revealed a positive non-linear relationship between UPFs intake and GDM risk (P_nonlinearity_ = 0.034, P_dose-response_ = 0.034, Fig. 3), but not for PE (P_nonlinearity_ = 0.696, P_dose-response_ = 0.812, Fig. 4). The non-linear dose-response analysis was not conducted for other outcomes due to insufficient studies.

### Sensitivity analyses and publication bias

Sensitivity analysis across the highest to the lowest meta-analysis for GDM, PE, PTB, LBW and SGA infants showed no significant influence of any single study (Supplemental Fig. 2).

No evidence of publication bias was found in articles related to the association with an increased risk of PE (*p* = 0.529, Egger’s test; *p* = 0.891, Begg’s), PTB (*p* = 0.458, Egger’s test; *p* = 0.473, Begg’s), LBW (*p* = 0.905, Egger’s test; *p* = 1.00, Begg’s test), and SGA infants (*p* = 0.348, Egger’s test; p = 1.00, Begg’s test). Although, for GDM, Egger’s test indicated potential publication bias (*p* < 0.001), not corroborated by Begg’s test (*p* = 0.241). As illustrated in Supplemental Fig. 3, the funnel plot was symmetrical for the association between the UPFs intake and all outcomes, except for studies that reported the risk of GDM disease.

### Quality of evidence

Utilizing the GRADE scale for quality evaluation, we detected the evidence for associations between UPFs intake and risk of GDM, PE, PTB, LBW and SGA infants was classified as moderate (Refer to Table 1).

## Discussion

In the realm of maternal and fetal health, the quality of dietary intake during pregnancy is of paramount significance. Accumulating evidence suggests a correlation between the consumption of UPFs and the deterioration of diet quality, potentially elevating the risk of various health complications [80–82]. This systematic review and meta-analysis aimed to elucidate the relationship between UPFs intake and adverse pregnancy outcomes including GDM, SGA, LBW, PTB, and PE, through an integrative analysis of existing studies. Our outcomes indicate a significant association between UPFs consumption and increased risks of PE, and GDM either prior to or during pregnancy. However, no significant association was found between UPFs intake and the risks of LBW, SGA, and PTB. Importantly, a 27% increment in the incidence of GDM was linked to a 100 g increase in UPF intake. Furthermore, a positive, non-linear relationship between UPF intake and GDM risk was identified through non-linear dose-response analysis, albeit no analogous association was found for PE.

The results of the current work showed a positive association between UPFs consumption and the risk of PE. In addition, subgroup analysis revealed this relationship to be more pronounced in studies using the NOVA-food classification and a Western dietary pattern for UPFs intake assessment, compared to those focusing on sweet intake. The NOVA classification categorizes foods based on the extent of processing, encompassing various UPFs. Moreover, the association between UPFs consumption and the risk of PE was significant in studies conducted in Asia (vs other regions). Prevalence of PE varies globally, ranging from 0.2–6.7% in Asia, 2.8–9.2% in Oceania, 2.8–5.2% in Europe, 2.6–4.0% in North America, and 1.8–7.7% in South America and the Caribbean [83]. However, the high heterogeneity in Asian studies should be noted when interpreting this result. Furthermore, a significant association was observed in studies involving women aged 30 years or older, aligning with the increased PE risk associated with advanced maternal age [84]. Additionally, a significant association was identified between PE risk and UPFs intake in women with pre-pregnancy BMI higher than 25 kg/m^2^ (vs BMI ≤25). This aligns with previous findings linking excessive weight gain in expectant mothers to an elevated PE risk, with overweight and obese mothers facing substantially higher risks [85].

The association between UPFs intake and PE can be elucidated through several mechanisms. The risk factors for PE, including GDM, maternal obesity, and advanced maternal age, are extensively documented in the literature [84–86]. It has been established that adopting healthy lifestyle habits (including dietary patterns) can mitigate these risk factors [87]. A higher intake of UPFs is associated with a diminished dietary quality, marked by an increased consumption of sugars and fats, alongside a decrease in fiber, protein, vitamins, and minerals [88, 89]. UPFs are known to contain elevated levels of pro-inflammatory agents such as refined sugars, salt, and trans fats. The ingestion of these inflammatory components can precipitate oxidative stress and systemic inflammation [90–92], which are implicated in the pathogenesis of preeclampsia [93, 94]. Additionally, the presence of trans fatty acids, added phosphates, and a high salt content in UPFs may impair endothelial function [95–97], a critical factor in the pathophysiology of hypertension observed in preeclampsia [98]. Furthermore, the intake of UPFs can alter the composition and diversity of the gut microbiota [99]. Studies have shown that food additives commonly found in UPFs, such as emulsifiers, sweeteners, and colorants, adversely affect the gut flora [100]. The interplay between the gut microbiota and the placenta, referred to as the “gut–placenta” axis, is crucial for understanding the etiology of PE. Dysbiosis of the gut microbiota and bacterial products like lipopolysaccharide (LPS) have been identified as promotive of PE [101, 102]. According to Kell et al., microbial infection, particularly through bacterial products such as LPS (also known as endotoxin), which is highly inflammatory, can initiate an innate immune response that exacerbates inflammation [103]. Hence, it is hypothesized that dysbiosis induced by UPFs consumption may play a significant role in the promotion of preeclampsia. Moreover, UPFs intake is positively associated with a risk of obesity [104], a condition marked by insulin resistance and hyperinsulinemia, crucial factors in PE development [105]. Pregnant women with obesity and PE exhibit higher leptin levels, correlating with increased Tumor Necrosis Factor-Alfa (TNF-α), Interleukin 6 (IL-6), and C-reactive protein concentrations [106, 107]. Additionally, excessive adipose tissue near the reproductive tract is the source of increased complement components and fragments in preeclamptic pregnancies. These complement proteins may promote an imbalance in angiogenic factors (characterized by increased production of antiangiogenic factors and a decrease in proangiogenic factors). This imbalance leads to placental injury, resulting in decreased blood flow to the tissue, and is accompanied by changes in cytokines levels (decreased IL-10 and increased TNF-α) before the onset of PE [108].

Our pooled analysis also revealed that higher UPFs intake was related to an increased risk of GDM. This association was significant in studies employing cohort and case-control designs (as opposed to those with cross-sectional methodologies). The inherent recall bias in cross-sectional studies that rely on self-reporting, is a notable limitation affecting the reliability of outcomes [109]. Moreover, this association was more pronounced in studies that used Western dietary patterns and fast-food consumption for the assessment of UPFs intake (vs those employing NOVA food classification and sweet consumption metrics). The concept of a dietary pattern, which represents the aggregate of eating and drinking habits, is critical as it exerts a greater impact on health and chronic diseases than any individual food item [110]. Additionally, the application of the NOVA food classification in existing studies is less frequent, suggesting the need for further research utilizing this methodology to derive more meaningful results. Geographical variations were also evident, with significant associations observed in studies conducted in America, compared to those in Asia and Europe. This is in context with the differing regional prevalences of GDM: 7.1% in North America and the Caribbean, 7.8% in Europe and 20.8% in South-East Asia [111]. Despite the higher prevalence of GDM in Asian populations, the greater intake of UPFs in American and European cohorts may have influenced the study outcomes [112–115]. Additionally, a positive association between UPFs intake and GDM risk was observed in studies focusing on women with a pre-pregnancy BMI > 25. Previous research indicates that being overweight or obese before and during pregnancy is a significant risk factor for GDM [116–118]. However, the scarcity of studies in women with pre-pregnancy BMI > 25 kg/m^2^kg/m suggests the need for more research in this demographic for robust conclusions.

Our outcomes also indicated that a 100 g increase in UPF intake was associated with a 27% increase in the risk of GDM. Moreover, the non-linear dose-response analysis similarly showed a positive, non-linear association between the consumption of UPFs and the risk of GDM. These findings underscore the significant impact that UPF consumption can have on GDM risk. The evidence points towards a robust and worrying correlation where even moderate increases in UPF intake can precipitate a marked rise in GDM risk, highlighting the critical need for dietary awareness and intervention among pregnant women. This aligns with broader nutritional science, emphasizing the importance of minimizing UPF consumption to mitigate not only GDM risk but potentially other metabolic disorders as well, given the multitude of adverse mechanisms through which UPFs affect glucose metabolism and insulin sensitivity.

Pathophysiologically, UPFs intake may increase GDM risk through several mechanisms. In pregnant women with GDM, pre-pregnancy reduced insulin sensitivity and β-cell dysfunction lead to hyperglycemia [119, 120]. The hypothesis that excessive sugar intake may augment body mass, thereby indirectly precipitating insulin resistance and subsequent diabetes, is widely recognized. Moreover, the liver’s capacity to assimilate and metabolize refined sugars prevalent in UPFs (such as fructose and sucrose) may be compromised, leading to augmented fat deposition and deteriorated insulin sensitivity [121]. Furthermore, insulin resistance may be induced by cosmetic ingredients present in UPFs. For example, dietary additives like carrageenan, employed as a thickening and stabilizing agent, may interfere with insulin signaling and thus foster insulin resistance [122]. Additionally, UPFs intake correlates with increased production of reactive oxygen species and inflammatory biomarkers [123], inducing insulin resistance through molecular pathways such as β-cell and mitochondrial dysfunction, decreased GLUT4 expression, impaired insulin signaling and heightened inflammatory responses [124]. Furthermore, UPFs often contain packaging materials like phthalates and bisphenol A, known to have endocrine disruption properties that may contribute to insulin resistance and diabetes development [125, 126]. The ingestion of substantial quantities of UPFs also elevates inflammation, a pivotal factor in the genesis of insulin resistance, culminating in hyperglycemia and the development of GDM [127]. A diet replete with saturated fats, trans fats, sugars, and salt, characteristic of high UPFs consumption, may contribute to chronic inflammation [128]. Furthermore, excessive UPFs consumption may supplant essential components of a balanced and nutritious diet. For instance, fruits and vegetables are associated with an anti-inflammatory effect [129]. In addition, the leaching of chemicals from food packaging into UPFs could introduce non-nutritional elements such as phthalates or bisphenol A, potentially eliciting an inflammatory response [130].

The present study did not establish a significant association between UPFs consumption and the risk of LBW. This result may be attributable to several factors. Firstly, a limited number of studies have evaluated the association between UPFs intake and LBW risk. Additionally, the intake of high-sugar foods (such as sugar-sweetened beverages) has been correlated with an increased risk of LBW in non-GDM subjects [34, 40]. This could be attributed to impaired fetal nutrition due to reduced vascular function, potentially induced by oxidative stress, inflammation, and endothelial dysfunction associated with high sugar consumption [131]. However, in GDM subjects this association may not be found due to the higher glucose loads in the fetus [47]. Therefore, additional research is warranted in both GDM and non-GDM populations to elucidate these relationships comprehensively.

Moreover, SGA was not associated with the intake of UPFs according to the pooled analysis of conducted studies. Although additional studies are necessary to explore this relationship further, existing evidence suggests that a fast-food dietary pattern may lead to increased fat intake and a reduced intake of essential micronutrients crucial for fetal development [132]. Maternal UPFs intake is linked to lower protein intake, reduced overall nutrition quality, and higher intake of trans fats, carbohydrates and saturated fats, which may increase the risk of neonatal adiposity [133–135]. Furthermore, higher fast-food intake during pregnancy has been linked with an elevated risk of maternal obesity, which in turn, may increase the likelihood of LGA babies [132, 136].

Regarding PTB, the current study found no association with UPFs consumption. Previous research has indicated that dietary patterns rich in fruits and vegetables are associated with a lower risk of PTB [37, 45]. Inadequate nutrition before and during pregnancy can lead to health issues for both the mother and fetus, increasing the risk of preterm delivery and intrauterine growth retardation [137]. The absence of an association between UPFs consumption and preterm delivery in this study could be due to various factors, including the need for a higher UPFs consumption threshold during pregnancy to manifest negative impacts on preterm birth. Additionally, the varied diet of pregnant women, typically including beneficial foods such as fruits, vegetables and nuts, may mitigate the adverse effects of UPFs.

The current investigation has several crucial strengths that make its findings highly significant. Firstly, by pooling all available observational data on the topic, the study provides a comprehensive and robust analysis of the relationship between UPFs intake and adverse pregnancy outcomes. Secondly, the study’s use of a dose-response analysis adds further weight to its conclusions and bolsters our understanding of the link between these two factors. However, there are limitations to consider. These include potential information and recall biases due to the self-reported nature of dietary intake assessments (such as the FFQ) and the absence of specific dietary tools for assessing UPFs consumption. Additionally, this meta-analysis included studies that did not use NOVA’s specialized dietary assessments. Moreover, dietary changes following pregnancy discovery could affect results, and the observational nature of the included studies precludes causal inference. Despite the inclusion of numerous confounding variables, several factors must be cautiously considered in the interpretation of the research findings. For instance, the socio-economic status of participants influences their dietary habits, while race and ethnicity may affect pregnancy outcomes. Furthermore, disparities in access to healthcare services can impact dietary choices and pregnancy outcomes. Other health statuses, such as mental health conditions among pregnant subjects, also influence dietary selections and pregnancy results [138, 139]. Finally, the availability of data on broader categories such as diabetes in pregnancy and hypertensive disorders was limited, hindering our ability to conduct a comprehensive analysis on these broader categories.

## Conclusion

Our outcomes indicate that prior to or during pregnancy, UPFs intake is associated with a higher risk of GDM and PE. However, no significant link tying UPFs intake to SGA, LWB and PTB was established. Importantly, a 100 g increment in UPFs intake was related to a 27% increase in GDM risk. This study aligns with global trends, where a rise in adverse pregnancy outcomes seems to align with the escalation of industrialization and the corresponding surge in UPFs production and consumption. Investigating the potential linkage between UPFs intake and the rise of adverse pregnancy outcomes may help in the development of nutrition-centric policies for expecting mothers and promote more health-conscious decision-making. To further substantiate these findings, extensive empirical research is required. Future studies should encompass observational research across diverse ethnic groups. Moreover, the adoption of more precise tools for measuring UPFs consumption is imperative. In observational research, it may be challenging to ascertain whether the consumption of UPFs directly contributes to adverse pregnancy outcomes or if it serves as an indicator of other underlying factors. Components of UPFs may escalate the risk of negative pregnancy outcomes. Moreover, UPF consumption could be part of a complex interplay affecting other variables that directly result in adverse outcomes. For instance, UPF intake could influence gestational weight gain, potentially leading to insulin resistance, which is known to correlate with unfavorable pregnancy outcomes, including GDM. Additionally, the consumption of UPFs may not only diminish dietary quality but also be linked with various lifestyle and dietary factors, such as poor diet quality, thereby increasing the risk of adverse pregnancy outcomes [140]. The bidirectional correlation between UPF consumption and unfavorable pregnancy outcomes also merits consideration. For example, individuals experiencing depression or other health conditions might alter their dietary patterns to include a higher intake of UPFs [138]. Evaluating changes in UPF consumption over time, utilizing precise questionnaires that assess food items classified as UPFs according to the NOVA food classification system, and their association with other health outcomes affecting pregnancy, such as obesity, could offer insights into this relationship. Considering these approaches is essential to enhance the depth and accuracy of investigations into the potential association between UPFs intake and the incidence of adverse pregnancy outcomes.

### Supplementary Information

**Supplementary Material 1.**

**Supplementary Material 2.**

**Supplementary Material 3.**

**Supplementary Material 4.**

**Supplementary Material 5.**

**Supplementary Material 6.**

**Supplementary Material 7.**

**Supplementary Material 8.**

## Data Availability

The datasets generated and/or analyzed during the current study are not publicly available due but are available from the corresponding author on reasonable request.
