# Supplementary material for: The association between ultra-processed food and common pregnancy adverse outcomes: a dose-response systematic review and meta-analysis
Source: BMC Pregnancy Childbirth. 2024 May 15;24:369. doi: 10.1186/s12884-024-06489-w (PMC11097443; doi:10.1186/s12884-024-06489-w)
Supplement: Supplementary file 3 — Supplementary Material 3. [file 12884_2024_6489_MOESM3_ESM.docx]

**
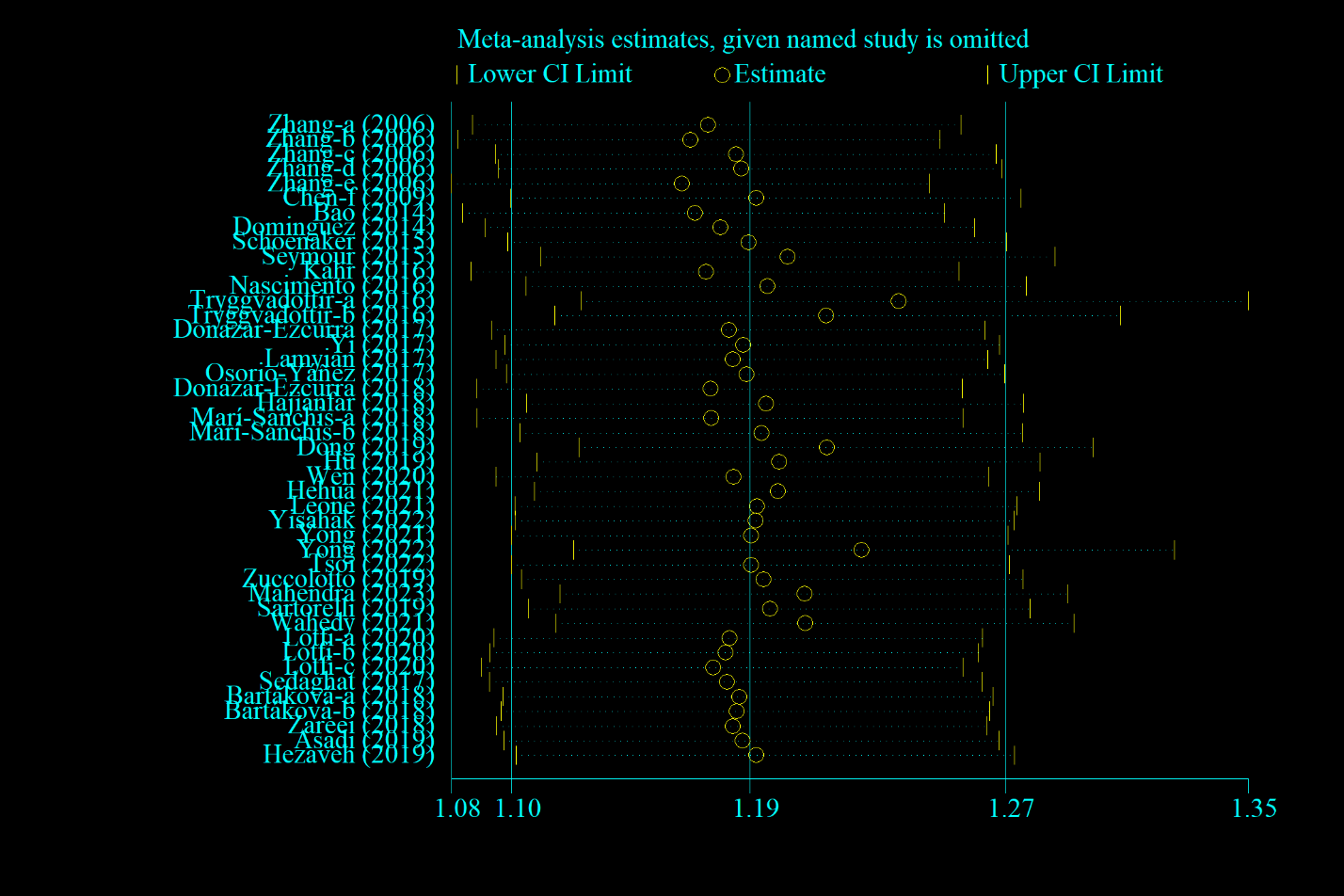
**

**Supplementary Figure 2-A.** Forest plots showing sensitivity analysis results of the relationship between ultra-processed foods intake and risk of gestational diabetes mellitus.


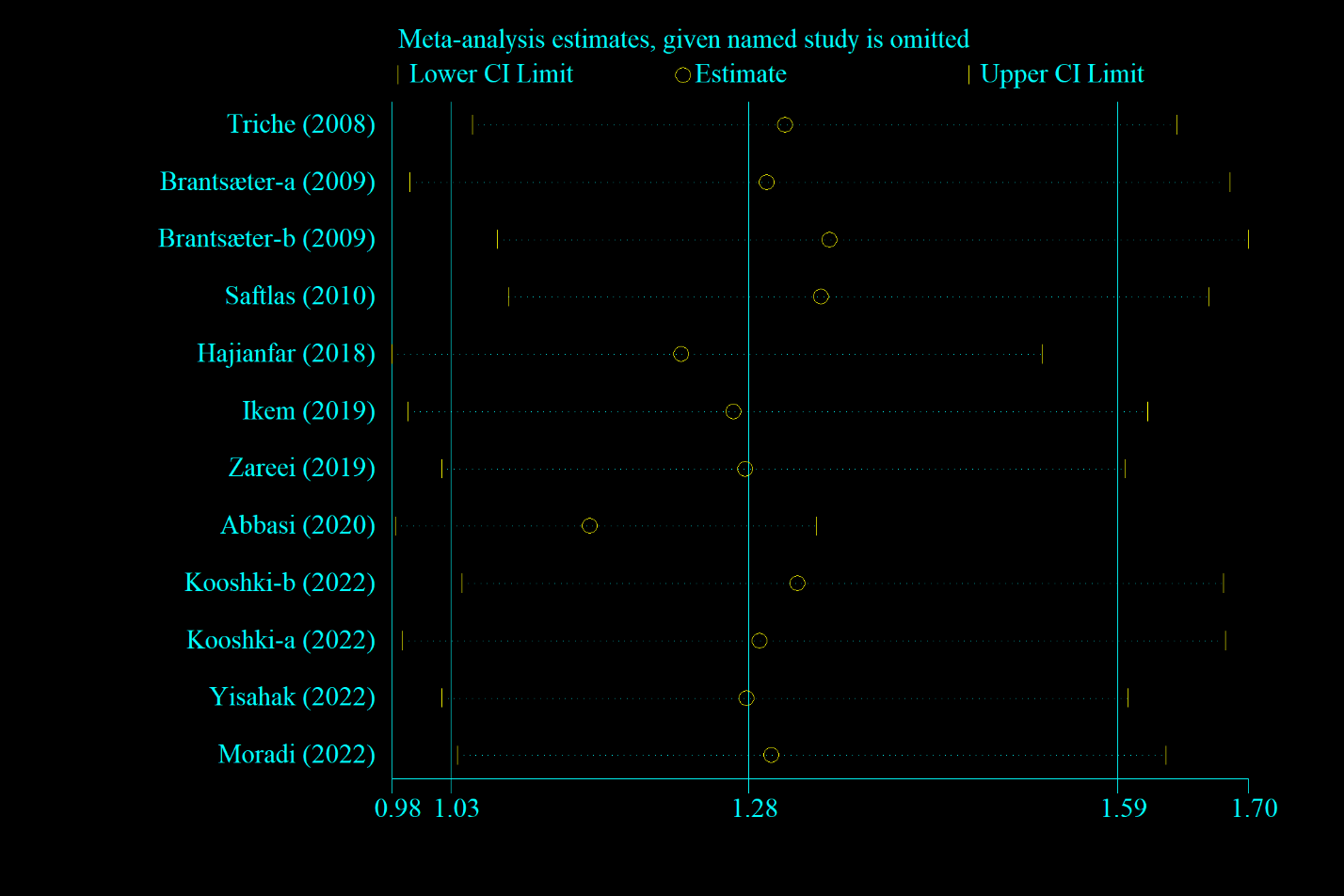


**Supplementary Figure 2-B.** Forest plots showing sensitivity analysis results of the relationship between ultra-processed foods intake and risk of preeclampsia.

**
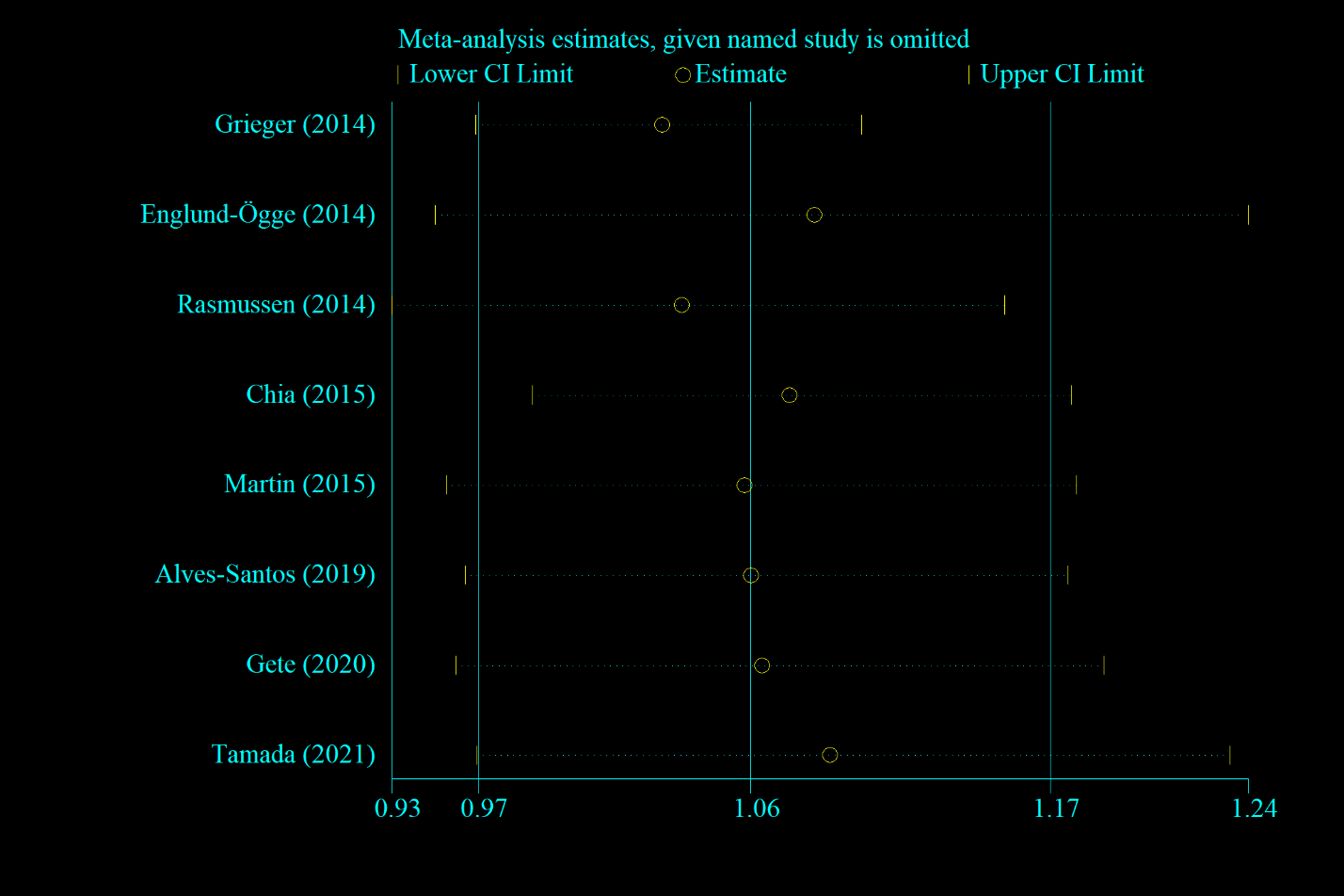
**

**Supplementary Figure 2-C.** Forest plots showing sensitivity analysis results of the relationship between ultra-processed foods intake and risk of preterm birth.


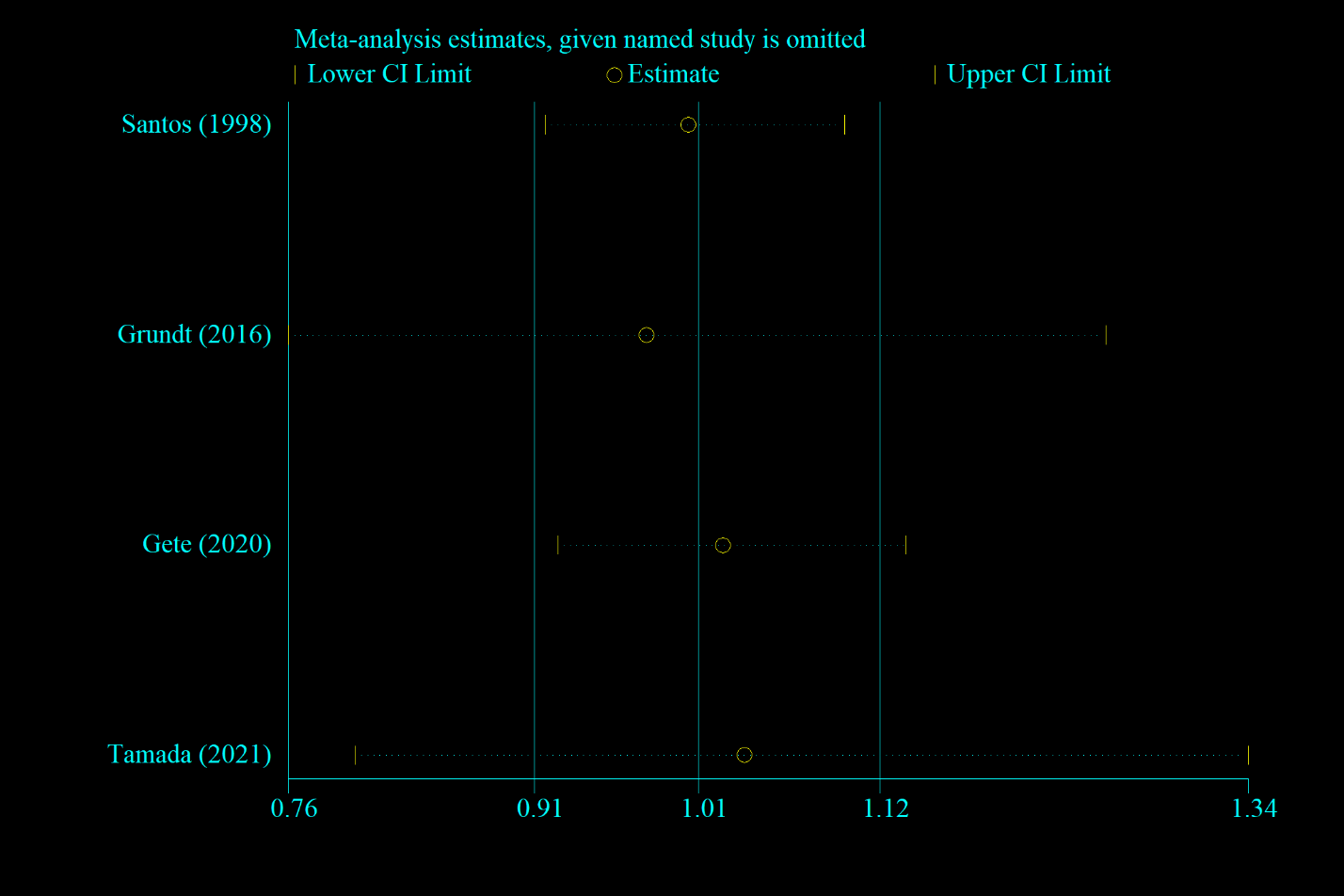


**Supplementary Figure 2-D.** Forest plots showing sensitivity analysis results of the relationship between ultra-processed foods intake and risk of low birth weight.


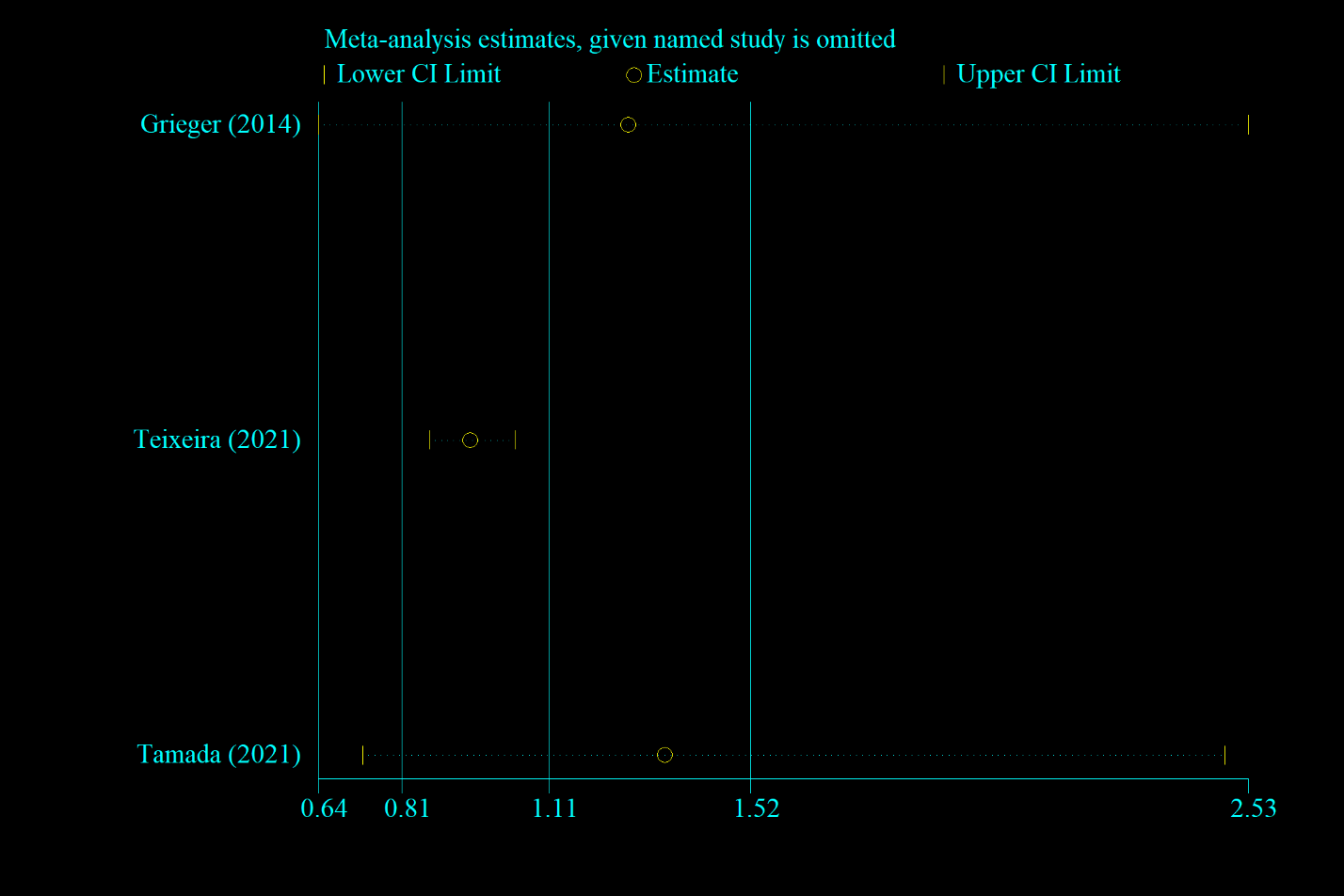


**Supplementary Figure 2-E.** Forest plots showing sensitivity analysis results of the relationship between ultra-processed foods intake and risk small for gestational age.
