# Supplementary material for: The association between ultra-processed food and common pregnancy adverse outcomes: a dose-response systematic review and meta-analysis
Source: BMC Pregnancy Childbirth. 2024 May 15;24:369. doi: 10.1186/s12884-024-06489-w (PMC11097443; doi:10.1186/s12884-024-06489-w)
Supplement: Supplementary file 4 — Supplementary Material 4. [file 12884_2024_6489_MOESM4_ESM.docx]

**
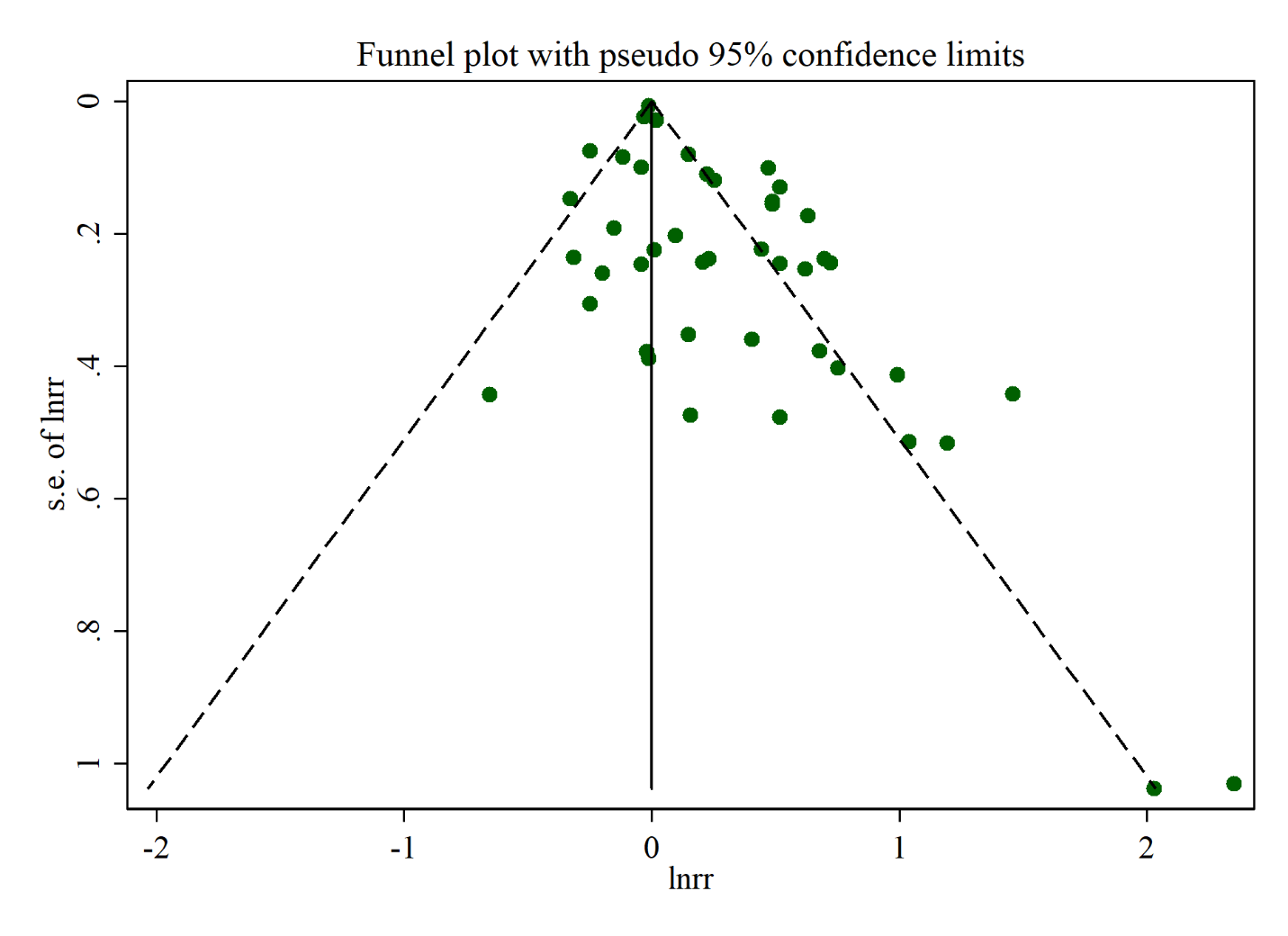
**

**Supplementary Figure 3-A.** Funnel plot for evaluation publication bias among studies reported risk of gestational diabetes mellitus.


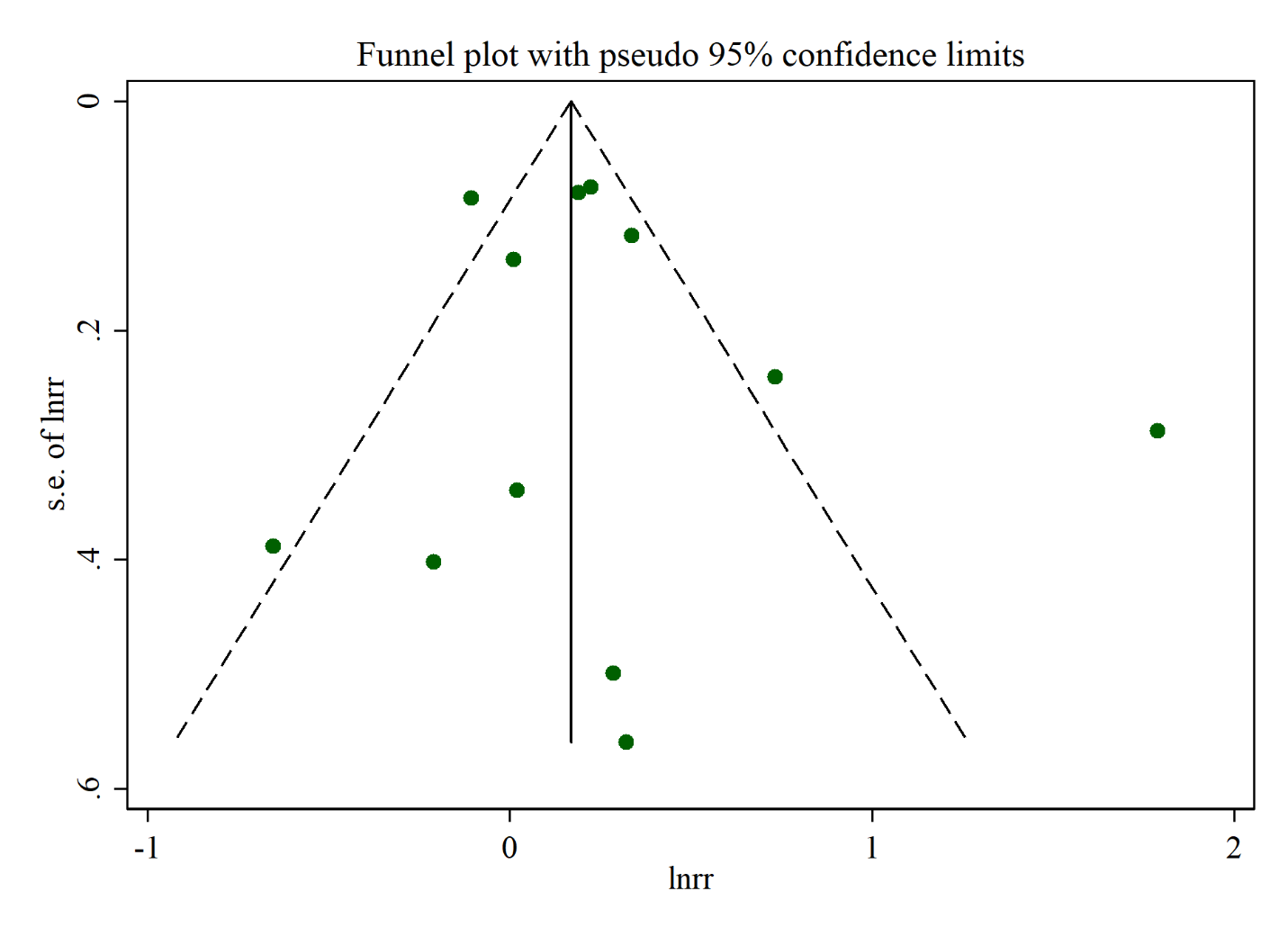


**Supplementary Figure 3-B.** Funnel plot for evaluation publication bias among studies reported risk of preeclampsia.


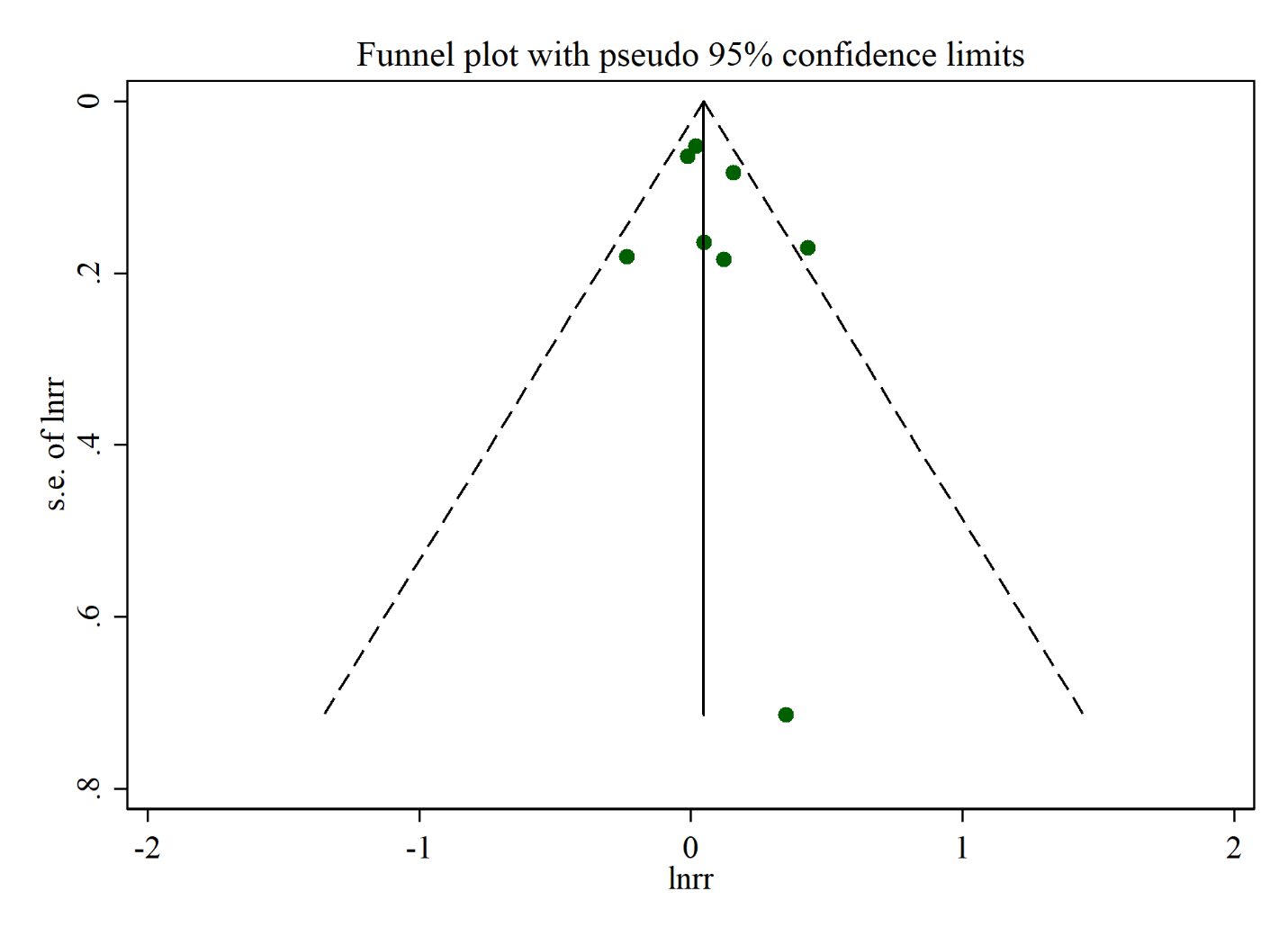


**Supplementary Figure 3-C.** Funnel plot for evaluation publication bias among studies reported risk of preterm birth.


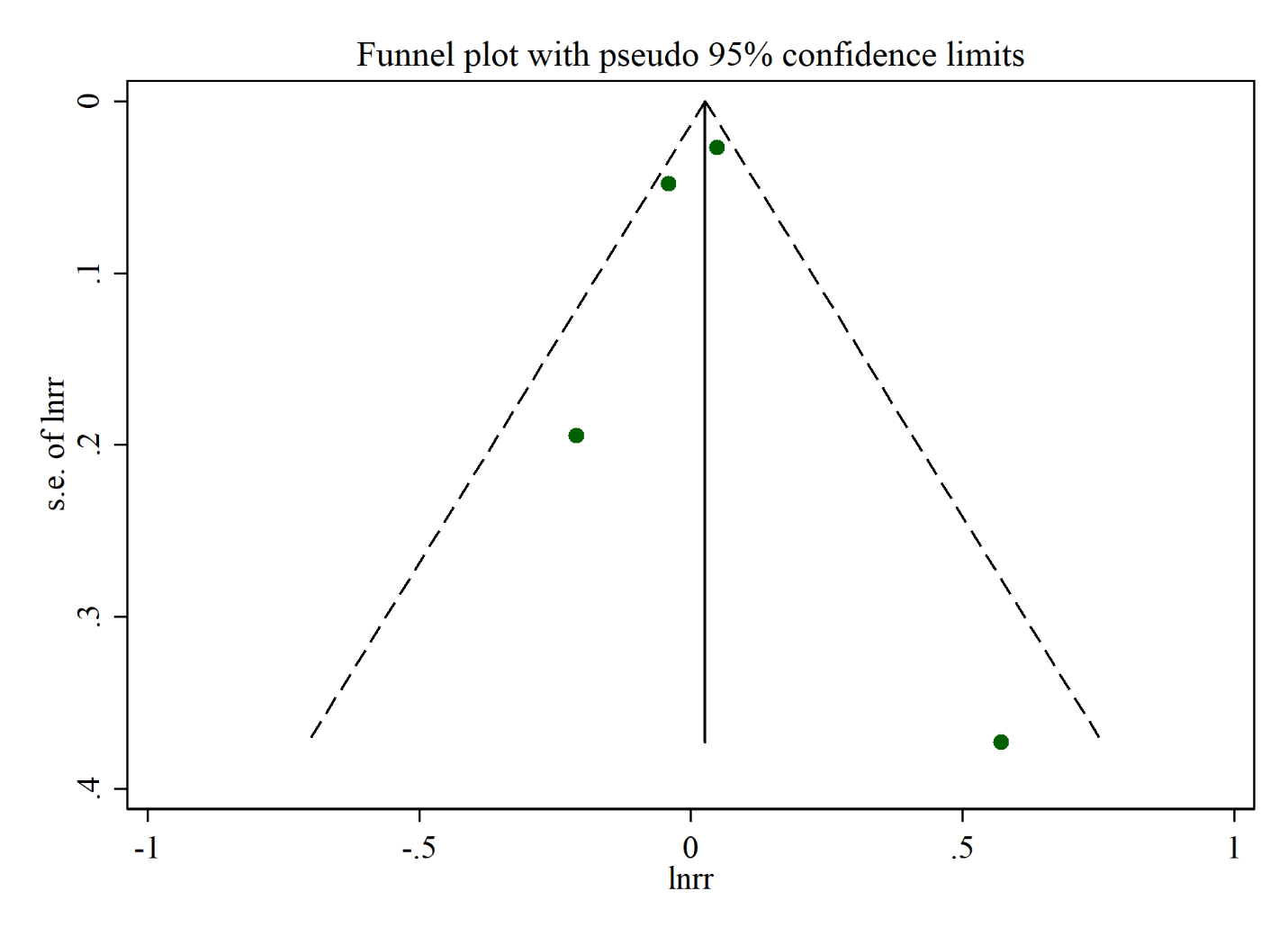


**Supplementary Figure 3-D.** Funnel plot for evaluation publication bias among studies reported risk of low birth weight.


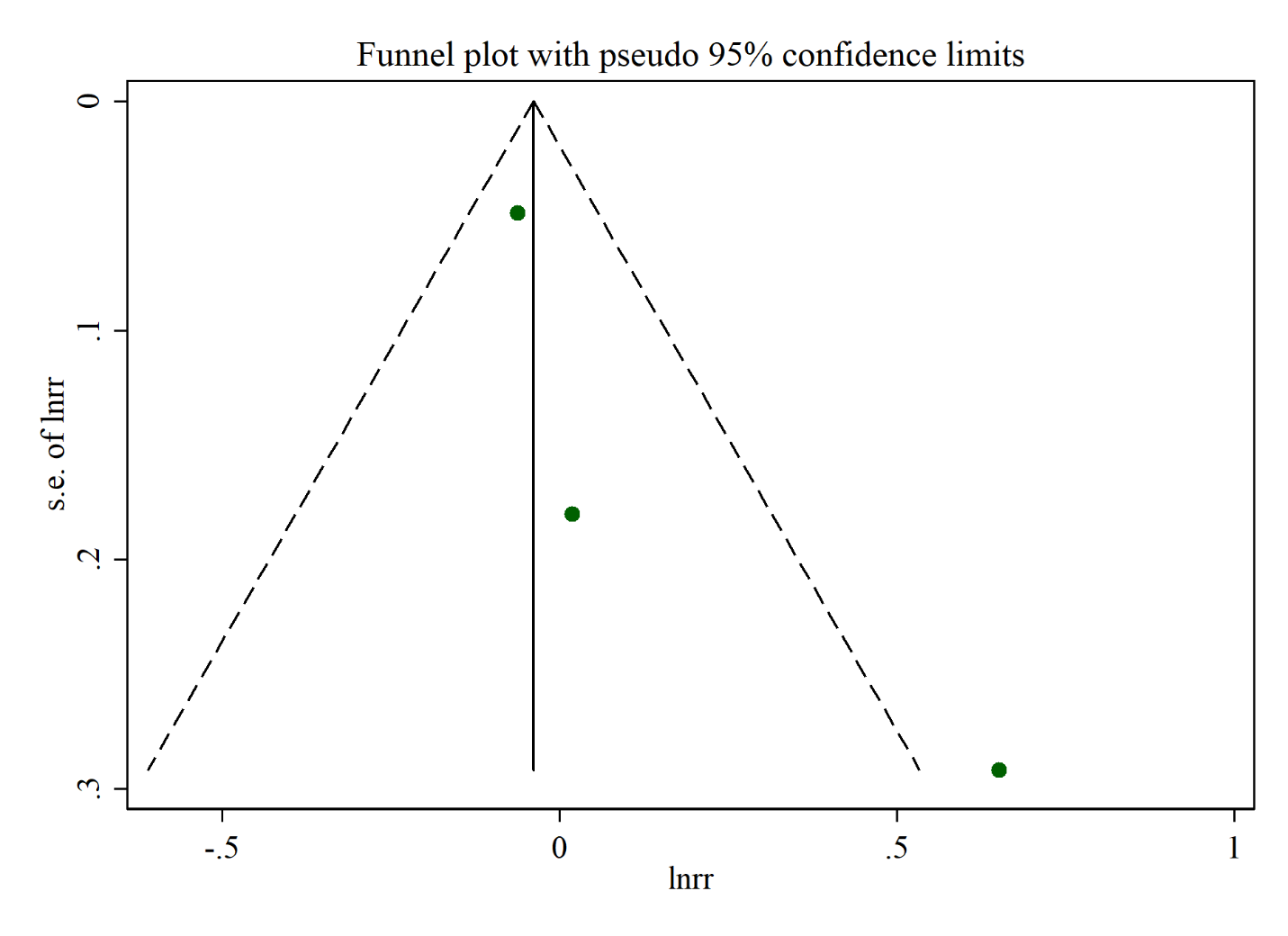


**Supplementary Figure 3-E.** Funnel plot for evaluation publication bias among studies reported risk of small for gestational age.
