# Supplementary material for: The association between ultra-processed food and common pregnancy adverse outcomes: a dose-response systematic review and meta-analysis
Source: BMC Pregnancy Childbirth. 2024 May 15;24:369. doi: 10.1186/s12884-024-06489-w (PMC11097443; doi:10.1186/s12884-024-06489-w)
Supplement: Supplementary file 5 — Supplementary Material 5. [file 12884_2024_6489_MOESM5_ESM.docx]

| **Supplementary Table 1.** Search strategies including the key terms and the queries for each database | |
| --- | --- |
| **Database**  **9/6/2023**  **September 6, 2023** | **key terms and the queries** |
| PubMed  (n=2098) | #1 "Fast Foods"[MeSH Terms] OR "Candy"[MeSH Terms] OR "Ice Cream"[MeSH Terms] OR "Chocolate"[MeSH Terms] OR "Snacks"[MeSH Terms] OR "Feeding Behavior"[MeSH Terms] OR "ultra-processed"[Title/Abstract] OR "ultraprocessed"[Title/Abstract] OR "Fast Foods"[Title/Abstract] OR "processed food"[Title/Abstract] OR "ultraprocessed food"[Title/Abstract] OR "ultra-processed food"[Title/Abstract] OR "processed meat"[Title/Abstract] OR "ultra-processed food"[Title/Abstract] OR "ham"[Title/Abstract] OR "sausages"[Title/Abstract] OR "hamburger"[Title/Abstract] OR "bacon"[Title/Abstract] OR "luncheon meats"[Title/Abstract] OR "ready-to-eat"[Title/Abstract] OR "ready-to-consume"[Title/Abstract] OR "industrialized"[Title/Abstract] OR "fast-food"[Title/Abstract] OR "fast-food"[Title/Abstract] OR "fast-food"[Title/Abstract] OR "junk food"[Title/Abstract] OR "prepared food"[Title/Abstract] OR "Candy"[Title/Abstract] OR "Ice Cream"[Title/Abstract] OR "Chocolate"[Title/Abstract] OR "Snacks"[Title/Abstract] OR "hot dog"[Title/Abstract] OR "burger"[Title/Abstract] OR "dietary patterns"[Title/Abstract] OR "dietary behaviors"[Title/Abstract] OR "dietary habits"[Title/Abstract] OR "NOVA"[Title/Abstract] OR "nova food*"[Title/Abstract] OR "NOVA food classification system"[Title/Abstract]  #2 "diabetes, gestational"[MeSH Terms] OR "gestational diabetes mellitus"[All Fields] OR "pregnancy induced diabetes"[All Fields] OR "gestational diabetes"[All Fields] OR "GDM"[All Fields] OR "Pre-Eclampsia"[MeSH Terms] OR "Eclampsia"[MeSH Terms] OR "hypertension, pregnancy induced"[MeSH Terms] OR "Preeclampsia"[All Fields] OR "Eclampsia"[All Fields] OR "gestosis eph"[All Fields] OR "pregnancy toxemia"[All Fields] OR "pregnancy-induced hypertension"[All Fields] OR "hypertensive disorders of pregnancy"[All Fields] OR "gestational hypertension"[All Fields] OR "pregnancy-associated hypertension"[All Fields] OR "pregnancy hypertension"[All Fields] OR "EPH Complex"[All Fields] OR "EPH Gestosis"[All Fields] OR "Pre-Eclampsia"[MeSH Terms] OR "Pre-Eclampsia"[All Fields] OR "Premature Birth"[MeSH Terms] OR "Fetal Growth Retardation"[MeSH Terms] OR "Premature Birth"[All Fields] OR "PTB"[All Fields] OR "Premature Birth"[All Fields] OR "fetal growth restriction"[All Fields] OR "FGR"[All Fields] OR "pre-term birth"[All Fields]  #3 #1 AND #2 |
| Web of Science (ISI)  (N=538) | #1 TOPIC: (“ultra-processed” OR “ultraprocessed” OR “fast foods” OR “processed food” OR “ultraprocessed food” OR “ultra-processed food” OR “processed meat” OR “ultra processed food” OR “ham” OR “sausages” OR “hamburger” OR “bacon” OR “luncheon meats” OR “ready-to-eat” OR “ready-to-consume” OR “industrialized” OR “fast-food” OR “fast food” OR “fast food” OR “junk food” OR “prepared food” OR “candy” OR “ice cream” OR “chocolate” OR “snacks” OR “hot dog” OR “burger” OR “dietary patterns” OR “dietary behaviors” OR “dietary habits” OR "NOVA" OR "nova food classify*" OR "nova food*" OR "nova food classif*"OR "NOVA food classification system")  #2 "diabetes, gestational" (Topic) or "gestational diabetes mellitus" (Topic) or "pregnancy induced diabetes" (Topic) or "gestational diabetes" (Topic) or "GDM" (Topic) or “Preeclampsia” (Topic) or “eclampsia” (Topic) or “gestosis, EPH” (Topic) or “pregnancy toxemia” (Topic) or “pregnancy-induced hypertension” (Topic) or “hypertensive disorders of pregnancy” (Topic) or “gestational hypertension” (Topic) or “pregnancy-associated hypertension” (Topic) not “pregnancy hypertension” (Topic) or “EPH Complex” (Topic) or “EPH Gestosis” (Topic) or “pre-term birth” (Topic) or “PTB” (Topic) or “Premature Birth” (Topic) or “fetal growth restriction” (Topic) or “FGR” (Topic)  #3 #1 AND #2 |
| Scopus  (N=832) | # 1 TITLE-ABS-KEY ( "ultra-processed food" ) OR TITLE-ABS-KEY ( "processed meat" ) OR TITLE-ABS-KEY ( "ultra processed food" ) OR TITLE-ABS-KEY ( "ham" ) OR TITLE-ABS-KEY ( "sausages" ) OR TITLE-ABS-KEY ( "hamburger" ) OR TITLE-ABS-KEY ( "bacon" ) OR TITLE-ABS-KEY ( "luncheon meats" ) OR TITLE-ABS-KEY ( "ready-to-eat" ) OR TITLE-ABS-KEY ( "ready-to-consume" ) OR TITLE-ABS-KEY ( "industrialized" ) ( TITLE-ABS-KEY ( "ultra-processed" ) OR TITLE-ABS-KEY ( "ultraprocessed" ) OR TITLE-ABS-KEY ( "fast foods" ) OR TITLE-ABS-KEY ( "processed food" ) OR TITLE-ABS-KEY ( "ultraprocessed OR TITLE-ABS-KEY ( "fast-food" ) OR TITLE-ABS-KEY ( "fast food" ) OR TITLE-ABS-KEY ( "fast food" ) OR TITLE-ABS-KEY ( "junk food" ) OR TITLE-ABS-KEY ( "prepared food" ) OR TITLE-ABS-KEY ( "candy" ) OR TITLE-ABS-KEY ( "ice cream" ) OR TITLE-ABS-KEY ( "chocolate" ) OR TITLE-ABS-KEY ( "snacks" ) OR TITLE-ABS-KEY ( "hot dog" ) OR TITLE-ABS-KEY ( "burger" ) OR TITLE-ABS-KEY ( "dietary patterns" ) OR TITLE-ABS-KEY ( "dietary behaviors" ) OR TITLE-ABS-KEY ( "dietary habits" ) OR TITLE-ABS-KEY ( "NOVA" ) OR TITLE-ABS-KEY ( "nova food classify*" ) OR TITLE-ABS-KEY ( "nova food*" ) OR TITLE-ABS-KEY ( "nova food classif*" ) OR TITLE-ABS-KEY ( "NOVA food classification system" ) ) )  #2 ( ( TITLE-ABS-KEY ( "diabetes, gestational" ) OR TITLE-ABS-KEY ( "gestational diabetes mellitus" ) OR TITLE-ABS-KEY ( "pregnancy induced diabetes" ) OR TITLE-ABS-KEY ( "gestational diabetes" ) OR TITLE-ABS-KEY ( "GDM" ) OR TITLE-ABS-KEY ( "Preeclampsia" ) OR TITLE-ABS-KEY ( "eclampsia" ) OR TITLE-ABS-KEY ( "gestosis, EPH" ) OR TITLE-ABS-KEY ( "pregnancy toxemia" ) OR TITLE-ABS-KEY ( "pregnancy-induced hypertension" ) OR TITLE-ABS-KEY ( "hypertensive disorders of pregnancy" ) OR TITLE-ABS-KEY ( "gestational hypertension" ) OR TITLE-ABS-KEY ( "pregnancy-associated hypertension" ) OR TITLE-ABS-KEY ( "pregnancy hypertension" ) OR TITLE-ABS-KEY ( "EPH Complex" ) OR TITLE-ABS-KEY ( "EPH Gestosis" ) OR TITLE-ABS-KEY ( "pre-term birth" ) OR TITLE-ABS-KEY ( "PTB" ) OR TITLE-ABS-KEY ( "Premature Birth" ) OR TITLE-ABS-KEY ( "fetal growth restriction" ) OR TITLE-ABS-KEY ( "FGR" ) ) )  #3 #1 AND #2 |
