# Supplementary material for: The association between ultra-processed food and common pregnancy adverse outcomes: a dose-response systematic review and meta-analysis
Source: BMC Pregnancy Childbirth. 2024 May 15;24:369. doi: 10.1186/s12884-024-06489-w (PMC11097443; doi:10.1186/s12884-024-06489-w)
Supplement: Supplementary file 6 — Supplementary Material 6. [file 12884_2024_6489_MOESM6_ESM.doc]

| **Supplemental Table 2.** Description of population, intervention, comparator and outcome (PICOs). | |
| --- | --- |
| **Population** | Adults (≥18 years) |
| **Intervention** | None |
| **Comparison** | Ultra-processed Food Intake |
| **Outcome** | The risk of pregnancy adverse outcomes including gestational diabetes mellitus (GDM), preeclampsia (PE), preterm birth (PTB), low birth weight (LBW) and small for gestational age (SGA) infants. |
| **Study design** | Observational research (cross-sectional, case-control, or cohort) |
