# Supplementary material for: The association between ultra-processed food and common pregnancy adverse outcomes: a dose-response systematic review and meta-analysis
Source: BMC Pregnancy Childbirth. 2024 May 15;24:369. doi: 10.1186/s12884-024-06489-w (PMC11097443; doi:10.1186/s12884-024-06489-w)
Supplement: Supplementary file 7 — Supplementary Material 7. [file 12884_2024_6489_MOESM7_ESM.docx]

| **Supplemental Table 3**: Reason for exclusion of retrieved articles | |
| --- | --- |
| References | Reason for exclusion |
| 1. Battarbee AN, Yee LM. Association of fast food and supermarket density with neonatal outcomes of pregnancies affected by gestational diabetes. American journal of perinatology. 2019 Nov;36(13):1405-11. 2. Dugas C, Perron J, Marc I, Weisnagel SJ, Robitaille J. Association between early introduction of fruit juice during infancy and childhood consumption of sweet-tasting foods and beverages among children exposed and unexposed to gestational diabetes mellitus in utero. Appetite. 2019 Jan 1;132:190-5. 3. Hu J, Aris IM, Lin PI, Wan N, Liu Y, Wang Y, Wen D. Association of maternal dietary patterns during pregnancy and offspring weight status across infancy: results from a prospective birth cohort in China. Nutrients. 2021 Jun 15;13(6):2040. 4. Huang L, Shang L, Yang W, Li D, Qi C, Xin J, Wang S, Yang L, Zeng L, Chung MC. High starchy food intake may increase the risk of adverse pregnancy outcomes: a nested case-control study in the Shaanxi province of Northwestern China. BMC pregnancy and childbirth. 2019 Dec;19(1):1-9. 5. Hajianfar H, Esmaillzadeh A, Feizi A, Shahshahan Z, Azadbakht L. Major maternal dietary patterns during early pregnancy and their association with neonatal anthropometric measurement. BioMed research international. 2018 May 31;2018. | Not relevant outcome |
| 1. Meltzer HM, Brantsæter AL, Nilsen RM, Magnus P, Alexander J, Haugen M. Effect of dietary factors in pregnancy on risk of pregnancy complications: results from the Norwegian Mother and Child Cohort Study. The American journal of clinical nutrition. 2011 Dec 1;94(suppl_6):1970S-4S. 2. Ramos-Leví AM, Pérez-Ferre N, Fernández MD, Del Valle L, Bordiu E, Bedia AR, Herraiz MA, Torrejón MJ, Calle-Pascual AL. Risk factors for gestational diabetes mellitus in a large population of women living in Spain: implications for preventative strategies. International journal of endocrinology. 2012 Oct;2012. 3. Eshriqui I, Vilela AA, Rebelo F, Farias DR, Castro MB, Kac G. Gestational dietary patterns are not associated with blood pressure changes during pregnancy and early postpartum in a Brazilian prospective cohort. European journal of nutrition. 2016 Feb;55:21-32. 4. Singh S, Urooj A. Influence of pre-pregnancy weight, food habits and lifestyle on gestational diabetes. Current Research in Nutrition and Food Science Journal. 2015 Aug 17;3(2):156-64. 5. Jenum AK, Sletner L, Voldner N, Vangen S, Mørkrid K, Andersen LF, Nakstad B, Skrivarhaug T, Rognerud-Jensen OH, Roald B, Birkeland KI. The STORK Groruddalen research programme: A population-based cohort study of gestational diabetes, physical activity, and obesity in pregnancy in a multiethnic population. Rationale, methods, study population, and participation rates. Scandinavian journal of public health. 2010 Nov;38(5_suppl):60-70. 6. Teixeira B, Cardoso M, Dias CC, Pereira-da-Silva L, e Silva D. Eating Habits During Pregnancy of Women Giving Birth Very Prematurely: An Exploratory Analysis. Acta Médica Portuguesa. 2023 Mar 6;36(6):401-7. | Without sufficient data |
| 1. Sengpiel V, Elind E, Bacelis J, Nilsson S, Grove J, Myhre R, Haugen M, Meltzer HM, Alexander J, Jacobsson B, Brantsæter AL. Maternal caffeine intake during pregnancy is associated with birth weight but not with gestational length: results from a large prospective observational cohort study. BMC medicine. 2013 Dec;11:1-8. 2. Rasmussen MA, Maslova E, Halldorsson TI, Olsen SF. Characterization of dietary patterns in the Danish national birth cohort in relation to preterm birth. PLoS One. 2014 Apr 18;9(4):e93644. 3. He JR, Yuan MY, Chen NN, Lu JH, Hu CY, Mai WB, Zhang RF, Pan YH, Qiu L, Wu YF, Xiao WQ. Maternal dietary patterns and gestational diabetes mellitus: a large prospective cohort study in China. British Journal of Nutrition. 2015 Apr;113(8):1292-300. 4. Shin D, Lee KW, Song WO. Dietary patterns during pregnancy are associated with risk of gestational diabetes mellitus. Nutrients. 2015 Nov 12;7(11):9369-82. 5. Mak JK, Pham NM, Lee AH, Tang L, Pan XF, Binns CW, Sun X. Dietary patterns during pregnancy and risk of gestational diabetes: a prospective cohort study in Western China. Nutrition journal. 2018 Dec;17(1):1-1. 6. Petry CJ, Ong KK, Hughes IA, Acerini CL, Dunger DB. Temporal trends in maternal food intake frequencies and associations with gestational diabetes: The Cambridge Baby Growth Study. Nutrients. 2019 Nov 19;11(11):2822. 7. Deepa R, Lewis MG, Van Schayck OC, Babu GR. Food habits in pregnancy and its association with gestational diabetes mellitus: results from a prospective cohort study in public hospitals of urban India. BMC nutrition. 2020 Dec;6:1-9. 8. Miele MJ, Souza RT, Calderon IM, Feitosa FE, Leite DF, Rocha Filho EA, Vettorazzi J, Mayrink J, Fernandes KG, Vieira MC, Pacagnella RC. Maternal nutrition status associated with pregnancy-related adverse outcomes. Nutrients. 2021 Jul 13;13(7):2398. 9. Wu W, Tang N, Zeng J, Jing J, Cai L. Dietary Protein Patterns during Pregnancy Are Associated with Risk of Gestational Diabetes Mellitus in Chinese Pregnant Women. Nutrients. 2022 Apr 13;14(8):1623. 10. Shin D, Lee KW, Song WO. Dietary patterns during pregnancy are associated with risk of gestational diabetes mellitus. Nutrients. 2015 Nov 12;7(11):9369-82. 11. Janevic T, Borrell LN, Savitz DA, Herring AH, Rundle A. Neighbourhood food environment and gestational diabetes in New York City. Paediatric and perinatal epidemiology. 2010 May;24(3):249-54. 12. Hu J, Li M, Li C, Yin S, Tao L, Li L, Wan N, Liu Y, Liu B, Zheng L, Wang X. Trimester-specific associations of maternal dietary patterns with preterm birth: China Medical University birth cohort study. Food & Function. 2023;14(16):7682-91. 13. Endeshaw M, Abebe F, Bedimo M, Asart A. Diet and pre-eclampsia: a prospective multicentre case–control study in Ethiopia. Midwifery. 2015 Jun 1;31(6):617-24. 14. Ancira‐Moreno M, O'Neill MS, Rivera‐Dommarco JÁ, Batis C, Rodríguez Ramírez S, Sánchez BN, Castillo‐Castrejón M, Vadillo‐Ortega F. Dietary patterns and diet quality during pregnancy and low birthweight: The PRINCESA cohort. Maternal & child nutrition. 2020 Jul;16(3):e12972. 15. Okubo H, Miyake Y, Sasaki S, Tanaka K, Murakami K, Hirota Y, Child Health Study Group. Maternal dietary patterns in pregnancy and fetal growth in Japan: the Osaka Maternal and Child Health Study. British journal of Nutrition. 2012 May;107(10):1526-33. | Not relevant exposure |
