# Supplementary material for: The association between ultra-processed food and common pregnancy adverse outcomes: a dose-response systematic review and meta-analysis
Source: BMC Pregnancy Childbirth. 2024 May 15;24:369. doi: 10.1186/s12884-024-06489-w (PMC11097443; doi:10.1186/s12884-024-06489-w)
Supplement: Supplementary file 8 — Supplementary Material 8. [file 12884_2024_6489_MOESM8_ESM.docx]

| **Supplementary Table 4. Characteristics of included studies.** | | | | | | | |
| --- | --- | --- | --- | --- | --- | --- | --- |
| **Quality score** | **Adjusted variables** | **Outcomes** | **Ultra-processed foods assessment method** | **Outcome criteria** | **Population/**  **Age/(Case)** | **Study design / Follow up**  **(years) / Source of data/ Health status** | **Author (year; location)** |
| +7/9 | Logistic regression for cigarette smoking, pregestational weight, skin color, and place of residence | no effect of caffeine on low birth  weight | 24 hours after delivery/ Chocolate bars/sweets | LBW:  low birth  weight (birth weight <2,500 g) | N = 1205/  (Case: 401) | Case-control studies | Santos et al. (1988, Brazil) |
| +9/9 | Age, BMI, race/ethnicity, cigarette smoking status, family history of diabetes in a first-degree relative, alcohol intake, physical activity and total energy | Higher intake of Western dietary patterns, processed meat, bacon, hot dogs, and sausage were associated with a higher risk of GDM | semi-quantitative food frequency questionnaire/ Western dietary pattern, Processed meat, Bacon, Hot dogs, Sausage | GDM:  diagnosis of GDM was based on self-reported information in the biennial questionnaire | N = 13,110/  Age = 31/ (Case: 758) | Cohort study /8 years/ NHS II/ pregnant women | Zhang et al. (2006, USA) |
| +8/9 | Smoking, BMI, clinic/private prenatal care provider, parity, race, maternal age, and education | chocolate consumption during  pregnancy may lower the risk of preeclampsia. | self-report questions in the first and third trimesters/ chocolate | PE:  NHLBI guidelines define preeclampsia | N = 1681/  Age = NR/ (Case: 63) | Cohort study /4 years/ pregnant women | Triche et al.  (2008, USA) |
| +9/9 | Additional adjustment for maternal age, maternal education, maternal height, maternal smoking, total energy intake, hypertension prior to pregnancy, dietary supplement use, dietary patterns and prepregnant BMI | High consumption of UPF increases the risk PE. However, a higher intake of Cakes and sweets was not significantly associated with the risk of PE | FFQ/ UPF, Cakes and sweets | PE: The diagnostic criteria for preeclampsia in Norway, according to guidelines issued by the Society for Gynecology, are blood pressure 140/90 after 20 wk of gestation, combined with proteinuria  .+1 dipstick on at least 2 occasions | N=23,423/ Age = NR/ (Case: 1267) | Cohort study /4 years/Norwegian Mother and Child Cohort Study (MoBa)/Nulliparous Pregnant Norwegian Women | Brantsæter et al. (2009, Norway) |
| +9/9 | Age, race/ethnicity, cigarette smoking status, family history of diabetes in a first-degree relative, alcohol intake, physical activity, BM1, western dietary pattern score | Pre-pregnancy higher consumption  of sugar-sweetened cola (5:5 servings/week) is associated with an elevated GDM risk,  whereas no significant association with GDM risk was observed for other SSBs and diet  beverages | FFQ/ Sugar-sweetened beverage | GDM:  medical record and self-reported | N=11475/ Age=31/ (Case:860) | Cohort study /10 years/ NHS II/ pregnant women | Chen et al. (2009, USA) |
| +8/9 | BMI and parity/abortion | Chocolate intake was associated with reduced odds of PE | self-report questions/ Chocolate | PE:  American College of Obstetricians and Gynecologists | N=11475/ Age=31/ (Case:58) | Cohort study /3 years/ Yale Health in Pregnancy Study | Saftlas et al. (2010, USA) |
| +9/9 | Age, parity, race/ethnicity, family history of diabetes, cigarette smoking and physical activity, total energy intake, diet quality, BMI | potential benefits of limiting fried food consumption in the prevention of GDM in women of reproductive age. | FFQ/ fried food | GDM:  medical record and self-reported | N=21079/ Age=31/ (Case:847) | Cohort study /10 years/ NHS II/ pregnant women | Bao et al. (2014, USA) |
| +9/9 | Age, total energy intake, smoking, physical activity, family history of diabetes, cardiovascular disease/hypertension at baseline, parity, adherence to Mediterranean dietary pattern score, alcohol intake, fiber intake, and sugar-sweetened soft drinks consumption, baseline BMI | pre-pregnancy higher consumption of fast food is an independent risk factor for gestational diabetes | FFQ/ Fast Food | GDM:  diagnosis of GDM made by a physician in the biennial questionnaire | N=3048/ Age=31/ (Case:159) | Cohort study /10.2 years/Seguimiento Universidad de Navarra | Dominguez et al. (2014, Spain) |
| +9/9 | Maternal age, pre-pregnancy body mass index, height, parity, total energy intake, maternal education, marital status, smoking, previous preterm delivery, household income, and the other dietary patterns | no independent association with preterm delivery for the Western pattern | FFQ/ “Western” | Preterm birth | N=66000/ Age=NR/ (Case:3505) | Cohort study /4 years/Norwegian Mother and Child Cohort Study/ pregnant women | Englund-Ögge et al. (2014, Norway) |
| +6/9 | Adjusted but not report | High fat/sugar/takeaway are associated with preterm delivery, shorter birth length, and earlier gestation. | FFQ/ High fat/sugar/takeaway (takeaway foods, potato chips, refined grains) | Preterm birth and SGA | N=309/ Age= 26.6 ± 5.4 | Cross-sectional study | Grieger et al. (2014, Australia) |
| +7/9 | adjusted | Western dietary pattern during early pregnancy was associated with preterm birth | FFQ/ Western | Preterm birth:  Danish Civil Registration System | N=60000/Age:29/ (Case: NR) | Danish National Birth Cohort | Rasmussen et al, (2014, Denmark) |
| +9/9 | Maternal age, race, maternal pre-pregnancy BMI status, educational level, household income, parity, marital status, smoking status, and energy intake. | Higher consumption of shellfish, pizza, salty snacks, candies, pancakes, tacos or burritos, and cakes or cookies were not associated with a decrease in the odds of preterm birth. | FFQ/ high factor loadings for shellfish, pizza, salty snacks, candies, pancakes, tacos or burritos, and cakes or cookies | Preterm birth | N=3143/ Age=NR/ (Case:364) | Prospective cohort study /9 years/ PIN (Pregnancy, Infection, and Nutrition) study | Martin et al. (2015, USA) |
| +9/9 | Age, total energy intake, parity, parous, hypertensive disorders of pregnancy, polycystic ovary syndrome, inter-pregnancy interval, smoking, physical activity, BMI | The ‘Meats, snacks, and sweets’ pattern was associated with a higher GDM risk | FFQ/ Meats, snacks and sweets | GDM:  self-reported physician diagnosis | N=3853/ Age= 28±1.4/ (Case:292) | Cohort study /9 years/Australian Longitudinal Study on Women’s Health (ALSWH) | Schoenaker et al. (2015, Australia) |
| +9/9 | Infant sex, birth order, maternal total energy intake and maternal age, ethnicity, prepregnancy BMI, weight gain until 26–28 wk of gestation, height, gestational diabetes mellitus status, educational status, alcohol use, smoking during pregnancy, and other dietary patterns. | No associations were observed for the processed meat in relation to birth outcomes. | 24-h recalls and 3-d food diaries/ processed meat | Preterm birth | N=923/ Age= 28.2 ±1.4/ (Case:70) | Cohort study /9 years/Growing Up in Singapore Towards  healthy Outcomes (GUSTO) | Chia et al. (2015, Singapore) |
| +7/9 | Energy intake, pregnancy BMI, birth order, smoking, alcohol intake, age, ethnicity, education, previous GDM, family history of diabetes, household monthly income, and other dietary patterns | Higher intake of processed meat did not significantly associate with risk of GDM | 24-h recalls/ processed meat | GDM:  OGTT | N=909/ Age= 33.5/(Case:160) | Cross-sectional study /Growing Up in Singapore Towards  healthy Outcomes (GUSTO) | Seymour et al. (2015, Singapore) |
| +8/9 | Gravidity, ethnicity, education, income, maternal age, body mass index, gestational age, preterm birth, gestational weight gain, and prior gestational diabetes | Higher intake of fast food was significantly associated with an increased risk of developing GDM | FFQ/ fast food | GDM:  OGTT | N=8912/ Age= 29.29/(Case:1152) | Cohort study / 5 year/PeriBank | Kahr et al (2016, USA) |
| +9/9 | Age, maternal height and pre‐pregnancy BMI, parity, education and income, diet patterns, i.e. three principal component variables, exercise, smoking, volume of alcohol intake per occasion prior to pregnancy, ASC intake, spontaneous labour, and offspring year of birth | High consumption of sugar‐sweetened carbonated soft drinks did not increase the risk of LBW. | FFQ/sugar‐sweetened carbonated soft drinks | LBW: (< 2500 g) | N= 50,280 /Age:28.9/ (Case: NR) | Norwegian Mother and Child Cohort Study (MoBa) Cohort study/ 6 years | Grundt et al. (2016, Norway) |
| +7/9 | Dietary patterns and body mass index, maternal age, maternal schooling, monthly income, family history of diabetes and parity | Western dietary pattern during early pregnancy was not associated with the development of GDM in this sample of Brazilian pregnant women with low income. | FFQ/ Western dietary pattern | GDM:  GDM diagnosis was based on the criteria developed by the IADPSG, after a 75 g, 2 h OGTT for GDM screening at a prenatal care visit at 24 to 28 gestational weeks; fasting >92mg/dl or 1 h >180 mg/dl or 2 h  >153mg/dl | N=841/ Age= 26.2 ± 5.8/ (Case:95) | Cohort study /3 years/ healthy pregnant women | Nascimento et al. (2016, Brazil) |
| +7/9 | Age, parity, pre-pregnancy weight, energy intake (kcal), weekly weight gain, and total MET | Higher intake of soft drinks and French fries were not associated with the risk of GDM | food record/ Soft drinks, French fries | GDM:  OGTT | N=168/ Age= 29.5/ (Case:4) | Cohort study /1.5 years/ National Bioethics Committee in Iceland / healthy pregnant women | Tryggvadottir et al. (2016, Iceland) |
| +9/9 | Age, baseline BMI, family history of diabetes, smoking status, physical activity, number of pregnancies before and multiple pregnancies | A positive association was found in the multivariable model between the highest quartile of adherence to Western dietary pattern and GDM incidence compared with the lowest quartile | FFQ/ Western dietary pattern | GDM:  Medical report | N=3455/ Age= 29.5/ (Case:173) | Cohort study /10.3 years/Seguimiento Universidad de Navarra/ healthy pregnant women | Donazar-Ezcurra et al. (2017, Spain) |
| +7/9 | BMI, history of GDM, family history of diabetes, third gestational weight gain, age, level of education, total energy intake, total fiber, and cholesterol intake | Higher intake of fast foods was significantly associated with an increased risk of GDM | FFQ/ fast food | GDM:  Pregnant women underwent a scheduled 100 g 3 h oral glucose tolerance test (OGTT) between the 24th–28th weeks of gestation. Diagnosis of GDM was based on the criteria set by the American  Diabetes Association. | N=1026/ Age= 26.7± 4.1/ (Case:71) | Cohort study/ 17-month/ Tehranian pregnant women | Lamyian et al. (2017, Iran) |
| +9/9 | Energy intake, maternal age, race/ethnicity, educational attainment, family history of diabetes, physical activity, alcohol, coffee, sugar-sweetened beverages, red and processed meats, calcium, dietary magnesium, and vitamin D intake, pre-pregnancy overweight status | Regular intake of total Fried Food was not associated with GDM risk. | FFQ/ Total Fried Food | GDM:  According to ADA 2004 guidelines, women were diagnosed with GDM if two or more 100-g, 3-  hr oral glucose tolerance test levels exceeded the following criteria: fasting ≥ 5.3 mmol/L (≥ 95  mg/dL); 1-hr ≥ 10.0 mmol/L (≥ 180 mg/dL); 2-hr ≥ 8.6 mmol/L (≥ 155 mg/dL); 3-hr ≥ 7.8 mmol/L (≥ 140 mg/dL) | N=3414/ Age= 33/ (Case:169) | Cohort study / 12 years/ Omega study | Osorio-Yáñez et al. (2017, USA) |
| +6/9 | Prepregnancy BMI, gestational age, physical activity, family history of diabetes, housing ownership | Adherence to the Western dietary pattern was associated with the risk of GDM | FFQ/ Western dietary pattern | GDM:  diagnostic testing was performed using a 100 g, 3-hour oral glucose tolerance test (OGTT). Women meeting the Carpenter and Coustan criteria, fasting 5.3mmol/l, 1 h 10.0mmol/l, 2 h 8.6mmol/l, and  3 h, 7.8mmol/l, were diagnosed with GDM (any two values  at or above established thresholds) | N = 388/  Age = 29.6/  (122 / 266) | Case-control studies | Sedaghat et al. (2017, Iran) |
| +9/9 | Maternal age, pre-pregnancy BMI, education, partner smoking, family history of diabetes, parity, daily food energy intake and physical activity | Adherence to the Western dietary pattern was not associate with the risk of GDM | 24-hour dietary recalls/ Western dietary pattern | GDM:  Between 24 and 28 gestational weeks, the GDM was screened by the 75-g oral glucose tolerance test (OGTT) | N=753/ Age= 28/ (Case:64) | Cohort study/ 1-7 year/Maternal and Child Health Care Hospital/ pregnant women | Yi et al. (2017, China) |
| +6/9 | No adjusted | Higher intake of sugar-sweetened beverage were associated with a higher risk of GDM, whereas no significant association with GDM risk was observed for goodies. | FFQ/ Goodies (sweet and  salty food), Sweet beverages | GDM:  GDM screening was carried out using OGTT with 75 g of  glucose performed between 24th and 30th week of pregnancy.  GDM diagnosis was established according to the  modified WHO criteria | N = 363/  Age = 29.6/  (Case:293) | Case-control studies | Bartáková et al. (2018, Czech) |
| +8/9 | Age. for total energy intake, BMI, family history of diabetes, current smoking status, total energy intake, physical activity, parity, fast-food consumption, Mediterranean dietary score, alcohol intake, multiple pregnancy, cardiovascular disease/hypertension at baseline, fiber intake, following special diet and snacking | Higher consumption of sweet beverages before pregnancy was an independent risk factor for GDM | FFQ/ Sweet beverages | GDM:  medical doctors | N=3396/ Age= 28/ (Case:172) | Cohort study /10.3 years/Seguimiento Universidad de Navarra/ healthy pregnant women | Donazar-Ezcurra et al. (2018, Spain) |
| +7/9 | Energy intake, age, BMI, socio-economic and physical activity | Adherence to the Western dietary pattern did not associate with the risk of GDM | FFQ/Western dietary pattern | GDM:  GDM was defined as the abnormal glucose homeostasis  including fasting plasma glucose concentration more than 95 mg/dL and 1-hour plasma glucose after eating 50 g of glucose, more than 140 mg/dL ) at 24-28 gestational weeks  PE:  Pre-eclampsia was  defined as a blood pressure of at least 140 mm Hg (systolic) or at least 90 mm Hg (diastolic) on at least two occasions and at least 4–6 hours apart after the 20th week of gestation in women known to be normo-tensive beforehand. Proteinuria was also defined as a protein concentration of 300 mg/L or more  in 24-h urine sample. | N=812/ Age= 30/ (Case: NR) | Cohort Study/ pregnant women | Hajianfar et al. (2018, Iran) |
| +8/9 | Age, BMI, family history of diabetes, parity, multiple pregnancy, smoking, physical activity, hypertension, adherence to Mediterranean diet, sugar-sweetened soft drinks, total energy intake, fiber intake, special diet, and snacking, heme iron | Higher intake of processed meat was significantly associated with risk of GDM. | FFQ/ Processed meat, Ham | GDM:  medical diagnosis | N=3298/ Age= 28.7±4.5/ (Case:172) | Cohort study /10.3 years/Seguimiento Universidad de Navarra/ healthy pregnant women | Marí‑Sanchis et al. (2018, Spain) |
| +6/9 | Age, educational level, BMI, weight changes in pregnancy, Number of deliveries, Getting Gestational Diabetes in previous pregnancy, Job, Physical activity | Adherence to an unhealthy dietary pattern was associated with the risk of GDM | FFQ/ unhealthy dietary pattern | GDM:  diagnose GDM is glucose challenge test (GCT) during 24–28ths weeks of pregnancy for all pregnant mothers under health care | N=204/ Age= 27.91±4.93/ (Case:104) | Case-control studies | Zareei et al. (2018, Iran) |
| +7/9 | Age, current smoker, alcohol consumption and first-trimester leisure physical activity | Higher intake of fast food was not associated with preterm birth | FFQ/ fast food | Preterm birth:  preterm birth (<37 gestational  weeks) | N=253/ Age= 26.7±5.5/ (Case:18) | Prospective cohort/ pregnant women | Alves-Santos et al. (2019, Brazil) |
| +7/9 | Age, BMI, family history of diabetes, history of foetal macrosomia, and age | Adherence to the Western dietary pattern was not associate with the risk of GDM | FFQ/ Western dietary pattern | GDM:  GDM diagnosis was approved according to the American Diabetic Association criteria | N=278/ Age= 29.0±5.17/ (Case:130) | Case-control studies | Asadi et al. (2019, Iran) |
| +8/9 | Age, BMI before pregnancy, depression, history of macrosomia babies, parity, smoking, drinking, education, occupation, household income, and physical activity, intakes of total meat, red meat, coffee, green tea, milk, soy isoflavone, magnesium, dietary fiber, dietary fat, saturated fat, snacks (potato chips and other crackers), and total energy intake | The highest quartile of chocolate consumption, compared with those in the lowest quartile, had a significantly lower risk of developing gestational diabetes | FFQ/ chocolate | GDM:  Cases were diagnosed using 75-gram oral glucose tolerance test according to the criteria of the Japan Diabetes Society | N=84948/ Age= 30.9±5.10/ (Case:1904) | Cohort study /3 years/ Japan Environment and Children’s Study | Dong et al. (2019, Japan) |
| +7/9 | Age, BMI, mother’s education, socioeconomic status, birth order of the child, and age of pregnancy | Higher intake of red and processed meat was not significantly associated with risk of GDM. | FFQ/ Red and processed meat | GDM:  GDM was defined as a fasting glucose level >95 mg/dL  and/or OGTT >155 mg/dL | N=320/ Age= 30.81±5.28/ (Case:152) | Case-control studies/ Arash Women's Hospital | Sajadi Hezaveh et al. (2019, Iran) |
| +8/9 | Pre-pregnancy BMI, age, parity, family income, education level, ethnicity, smoking status, total energy intake from three-day food diaries, and physical activity | Higher intake of sweet foods was not significantly associated with risk of GDM. | FFQ/Sweet foods | GDM:  GDM was diagnosed with a 75 g 2-h oral glucose tolerance test at 24–28 weeks of gestation | N=1014/ Age= NR/ (Case:238) | Prospective Cohort Study in China | Hu et al. (2019, China) |
| +7/9 | Pre-pregnancy BMI, parity, maternal age, smoking status, physical activity in pregnancy, sociodemographic status of pair, history of hypertension and total energy intake, and maternal height | Adherence to the Western dietary pattern was associated with the risk of PE | FFQ/ Western dietary pattern | PE:  Pre-eclampsia and severe PE were defined based on doctor's diagnosis retrieved from the Danish National Patient Registry | N=55139/ Age= 30.0 ± 4.3/ (Case:1168) | Cohort study/ Danish National Birth Cohort | Ikem et al. (2019, Denmark) |
| +7/9 | Age, gestational week at the time of the interview, schooling, smoking, physical activity, total energy intake, BMI adequacy, parity, gestational diabetes mellitus history, and family history of diabetes mellitus | Higher intake of UPF was not significantly associated with the risk of GDM. | 24-hour dietary recalls/UPF | GDM:  diagnosis was based on the 2014 World Health Organization (WHO) criteria | N=785/ Age= 28/ (Case:139) | Cross-sectional study / Unified Health System of Ribeirão Preto | Sartorelli et al. (2019, Brazil) |
| +6/9 | BMI, weight changes in pregnancy, number of deliveries, getting gestational diabetes in previous pregnancy, occupational status, and physical activity | Adherence to an unhealthy dietary pattern was not associated with the risk of PE | FFQ/ unhealthy dietary pattern | PE:  diagnosis of preeclampsia  was blood pressure 190/140 mmHg after 30 minutes of  rest in two different positions with proteinuria of 30mg/dL  (+ 1 on dipstick) in randomized urine specimens in the case  of lack of urinary tract infection or 24-hour urine protein  the excretion rate of 300 mg, approved by the lab and the attending physician on the wards | N=182/ Age= 28.96 ± 5.85/ (Case:82) | Cross-sectional study / Fasa Vali-e-Asr Hospital | Zareei et al. (2019, Iran) |
| +7/9 | Age, gestational week at the time of the interview, schooling, smoking, physical activity, total energy intake, BMI adequacy, parity, gestational diabetes mellitus history, and family history of diabetes mellitus | Adherence to a snack dietary pattern was not associated with the risk of GDM. | 24-hour dietary recalls/Snacks pattern | GDM:  diagnosis was based on the 2014 World Health Organization (WHO) criteria | N=785/ Age= 28/ (Case:139) | Cross-sectional study / Unified Health System of Ribeirão Preto | Zuccolotto et al. (2019, Brazil) |
| +6/9 | Adjusting for Confounders | Higher intake of sugars, fast food, and fried food was significantly associated with the risk of GDM. | 24-hour food recall/ sugars, fast food, fried food | GDM: NR | N=341/ Age= 30/ (Case:173) | Case-control studies | Lotfi et al. (2020, Iran) |
| +6/9 | BMI, age, gestational age, physical activity, parity, history of miscarriage, drug use and differences in physical activities prior to pregnancy as opposed to during the pregnancy | Adherence to the Western dietary pattern was associated with the risk of PE | FFQ/ Western dietary pattern | PE:  Preeclampsia cases were diagnosed using criteria such as a blood pressure above 140/90, a 24-h proteinuria  >300 mg, and a health and hospital record confirmed by a gynecologist in case of hospitalization | N=510/ Age= 25/ (Case:170) | Case-control studies | Abbasi et al. (2020, Iran) |
| +9/9 | Alcohol intake, physical activity, GDM, HDP, and pre-pregnancy BMI | adherence to the  Sugar, refined grain, and processed food patterns before pregnancy are not associated with a lower risk of preterm birth and LBW | FFQ/ Sugar, refined grain, and processed foods | preterm birth, defined as a live birth before 37 weeks of gestation.  LBW was defined as an infant whose birth weight is <2500  g, regardless of gestational age. | N=3422/ Age= 32.1 ± 3.2/ (Case:271) | Cohort study /12 years/ Australian Longitudinal Study on Women’s Health (ALSWH) | Gete et al. (2020, Australia) |
| +7/9 | Pre-pregnancy BMI, age, parity, family income, education level, ethnicity, smoking status, total energy intake from three-day food diaries, and physical activity | Adherence to the Sweets-based pattern was not associated with the risk of GDM | FFQ/ Sweets-based pattern | GDM:  Perinatal outcomes other than GDM were obtained from medical records | N=324/ Age= NR/ (Case:101) | Cohort Study / Longitudinal Twin Study (LoTiS) | Wen et al. (2020, China) |
| +7/9 | Age and BMI, race, education level, family income, physical activity, family diabetes history, parity, smoking and drinking status, the presence of hypertension/cardiac diseases, eclampsia/polycystic ovarian syndrome, or depression or other chronic diseases; and total energy intake and total energy intake based on model1 | Adherence to the Sweets-based pattern was not associated with the risk of GDM | FFQ/ Sweets-based pattern | GDM:  GDM was diagnosed based on the results of the 75-g oral glucose tolerance test completed during 24–28 weeks of gestation according to  the 2010 recommendations of the International Association of Diabetes and Pregnancy Study Groups | N=2244/ Age= 31.67/ (Case:476) | Cohort Study/ Northeast Cohort Study of China | Hehua et al. (2021, China) |
| +9/9 | Age at registration, smoking histories of second/third trimester, maternal educational status, annual income, alcohol intake of second/third trimester, In vitro fertilization, maternal BMI, histories of pregnancy loss, parity, working hours, energy intake, hypertensive disorders of pregnancy, gestational diabetes, and each dietary habits | A higher intake of beverages had not a higher risk of Preterm birth, SGA and LBW. | Questions /Ready-made meals | Preterm birth: (< 37 wk gestation)  SGA:  LBW: (< 2500 g) | N=94062/ | Japan Environment and Children Study (JECS), Cohort study/ 3 years | Tamada et al. (2021, Japan) |
| +8/9 | Age, BMI, education, smoking status, physical activity, family history of diabetes, recruitment year, time between recruitment and the first pregnancy or GDM, number of pregnancies during follow-up, parity, multiple pregnancies, time spent watching TV, hypertension, following a nutritional therapy, and energy intake. | Higher intake of UPF was not significantly associated with the risk of GDM. | FFQ/ UPF | GDM:  medical diagnosis | N=3730/ Age= 26/ (Case:186) | Cohort study /10.3 years/Seguimiento Universidad de Navarra/ healthy pregnant women | Leone et al. (2021, Spain) |
| +7/9 | Adjusted by the other dietary patterns | the highest quintile of the DP 'Snacks, sandwiches, sweets, and soft drinks' were significantly more likely to deliver SGA babies | FFQ/ Snacks, sandwiches, sweets and soft drinks | SGA:  Newborns were classified as SGA if their weight and/or length, adjusted by gestational age and sex, were below the 10th percentile of the INTERGROWTH-21st standards. | N=299/ Age= 25.9/ (Case:78) | Case-control / ProcriAr Cohort Study | Teixeira et al. (2021, Brazil) |
| +6/9 | family history of diabetes mellitus, family history of cardiovascular diseases, family history of hyperlipidemia, physical activity (Total MET), gestational weight gain (kg), and fasting plasma glucose | Adherence to an unhealthy dietary pattern was not associated with the risk of GDM | FFQ/ unhealthy dietary pattern | GDM:  WHO criteria | N=210/ Age= 27/ (Case: 70) | Case-control studies | Wahedy et al. (2021, Palestine) |
| +8/9 | Age, parity, total energy, pre-pregnancy BMI, and total GWG | The highest tertile of processed meat intake  in the second trimester was not positively associated with the GDM risk | FFQ/ Processed meat | GDM:  World Health Organization 2013 criteria | N=452/ Age= 30.01/ (Case:48) | Cohort study /2 years/ The Seremban Cohort Study (SECOST) | Yong et al. (2021, Malaysia) |
| +8/9 | Age, smoking habit, family income, education level, marital status, energy intake, delivery method, other dietary patterns | higher intake of Chocolate drinks was not associated with an increased risk of GDM | FFQ/ Chocolate drinks | GDM:  GDM diagnosis, outlined in the Perinatal Care Manual Third Edition, was based on a standard two-point diagnostic 75 g oral glucose tolerance test (OGTT) performed between 28 and 32 weeks of gestation. | N=452/ Age= 30.01/ (Case:48) | Cohort study /2 years/ The Seremban Cohort Study (SECOST) | Yong et al. (2022, Malaysia) |
| +6/9 | BMI, age, residence, and educational level | Higher intake of sugars and sweets was related to the odds of PE. However, higher consumption of sweet beverages was not associated with PE. | FFQ/Sugars and sweets  Sugar-sweetened beverage | PE:  diagnosis of preeclampsia by a physician | N=228/ Age= 30.59± 6.53 / (Case: 100) | Case-control studies/Sabzevar Shahidan Mobini Hospital | Kooshki et al. (2022, Iran) |
| +6/9 | No adjusted | Increased frequency of fast foods, carbonated soft drinks, and table salt were also associated with the incidence of PE. | FFQ/ carbonated soft drinks | PE:  The diagnosis of preeclampsia was done based on systolic blood pressure equal to or above 140 mmHg and/or diastolic blood pressure equal to or above 90 mmHg, with proteinuria greater than  300 mg in the 24-h urine or equal to or greater than +1 in the strip test. | N=240/ Age= 32± 10.5 / (Case: 90) | Case-control studies | Moradi et al. (2022, Iran) |
| +7/9 | Age, smoking habit, family income, education level, marital status, energy intake, delivery method, other dietary patterns | Adherence to a sweet and fast-food pattern was not associated with the risk of GDM | FFQ/ Sweet and fast-food pattern | GDM:  World Health Organization 2013 criteria | N=156/ Age= 32.7 / (Case: 21) | Cohort study /1 year/ Prince of Wales Hospital/pregnant women in Hong Kong | Tsoi et al. (2022, China) |
| +8/9 | Age, race/ethnicity, education, marital status, nulliparity, pre-pregnancy BMI, moderate to vigorous physical activity, sleep duration, and total energy intake | Higher intake of UPF was not significantly associated with the risk of PE and GDM. | FFQ/ UPF | PE: physician  GDM: GDM was defined based on Carpenter and Coustan criteria, as endorsed by the American College of Obstetricians and Gynecologists and the American Diabetes Association | N=1948/ Age= 27.5 / (Case: 63) | Cohort study /4 yearS/ prospective U.S. cohort | Yisahak et al. (2022, USA) |
| +7/9 | Adjusted | A higher intake of fried and fast food had a lower GDM. | FFQ/ fried and fast food | GDM:  Gestational diabetes was assessed by a 75 g oral glucose tolerance test at 24–28 weeks’ gestation, applying 2013 WHO criteria. | N=785/ Age= 27.5 / (Case: NR) | Cohort study / Bangalore Nutrition Gestational diabetes LiFEstyle Study (BANGLES) | Mahendra et al. (2023, UK) |
| **Abbreviations:** GDM; Gestational Diabetes Mellitus, PE; Preeclampsia, LBW; Low birth weight, SGA; Small for gestational age, NHLBI; National Heart, Lung and Blood Institute, BMI; body mass index, FFQ; food-frequency questionnaire, UPF; ultra-processed food, NHS II; Nurses' Health Study II, OGTT; Oral Glucose Tolerance Test. | | | | | | | |
