# Supplementary material for: The association between ultra-processed food and common pregnancy adverse outcomes: a dose-response systematic review and meta-analysis
Source: BMC Pregnancy Childbirth. 2024 May 15;24:369. doi: 10.1186/s12884-024-06489-w (PMC11097443; doi:10.1186/s12884-024-06489-w)
Supplement: Supplementary file 8 [file 12884_2024_6489_MOESM1_ESM.docx]

Data extraction form (supplement file)

ID	Author	Country	Year	Follow up	studyt	articipant	regnancy	case	age	esment to	nancy or	ase ascertain	rr	lci	uci	study cohort	expoxure
1	Zhang-a	USA	2006	8	2	13110	23	758	31	ffq	early	self-reported	1.63	1.2	2.21	Nurses_ Health	Western
2	Zhang-b	USA	2006	8	2	13110	23	758	31	ffq	early	self-reported	1.68	1.3	2.16	Nurses_ Health	Process meat
3	Zhang-c	USA	2006	8	2	13110	23	758	31	ffq	early	self-reported	1.29	1.02	1.63	Nurses_ Health	Bacon
4	Zhang-d	USA	2006	8	2	13110	23	758	31	ffq	early	self-reported	1.25	1.01	1.56	Nurses_ Health	Hot dogs
5	Zhang-e	USA	2006	8	2	13110	23	758	31	ffq	early	self-reported	1.6	1.31	1.95	Nurses_ Health	Sausage
6	Chen	USA	2009	10	2	13475	23	860	31	FFQ 133	early	medical record	1.16	0.99	1.36	Nurses_ Health	SSB
7	Bao	USA	2014	10	2	21,079	23.7	847	31	FFQ 134	early	medical record	1.88	1.34	2.64	Nurses_ Health	fried food
8	Dominguez	Spain	2014	10.2	2	3048	21.4	159	28.9	136-item s	early	physician	1.86	1.13	3.06	SUN Project	Fast Food
9	Schoenaker	Austra	2015	9	2	3,853	24	292	28	FFQ 101	Pre-pregn	self-reported	1.23	0.76	1.97	Australian Lon	Meats, snacks and sweets
10	Seymour	Singapore	2015		3	909	26	160	31.5	24-h dieta	Midpregna	OGTT	0.96	0.79	1.17	GUSTO	Pasta-cheese-processed-meat-diet
11	Kahr	USA	2016		2	8,912		1152	29.29			OGTT	1.63	1.21	2.19	(PeriBank)	Fast food
12	Nascimento	Brazil	2016	3	2	841		95	26.2	FFQ	Midpregna	OGTT	0.78	0.43	1.43	Brazil - a cohor	Western
13	Tryggvadottir-a	Iceland	2016	1.5	2	168		4	29.5	food recor	Midpregna	OGTT	0.99	0.98	1.01	National Bioethi	Soft drinks
14	Tryggvadottir-b	Iceland	2016	1.5	2	168		4	29.5	food recor	Midpregna	OGTT	1.02	0.99	1.11	National Bioethi	French fries
15	Donazar-Ezcurra	Spain	2017	10.3	2	3455	24.1	173	29	ffq	Pre-pregn	medical report	1.56	1.01	2.43	SUN	Western dietary pattern
16	Yi	China	2017	1	2	753	21.3	64	28	24-hour di	early	OGTT	1.68	0.66	4.29	Maternal and Ch	Western pattern
17	Lamyian	Iran	2017	1	2	1026	25.4	71	26.7	ffq 168	Pre-pregn	OGTT	2.12	1.12	5.43	iran	Total fast food
18	Osorio-Yáñez	USA	2017	12	2	3414	23.5	169	33	ffq 121	early	OGTT	1.26	0.79	2.01	Omega Cohort S	fried food
19	Donazar-Ezcurra	Spain	2018	10.3	2	3396	21.4	172	28	ffq 136	early	OGTT	2.06	1.28	3.34	SUN	sugar-sweetened soft drink (SSSD)
20	Hajianfar	Iran	2018		2	812				ffq 117	early	OGTT	0.52	0.22	1.25	iran, isfahan	western
21	Marí-Sanchis-a	Spain	2018	10.3	2	3298	21.4	172	28	ffq 136	Pre-pregn	OGTT	2.01	1.26	3.21	SUN	Processed meat
22	Marí-Sanchis-b	Spain	2018	10.3	2	3298	21.4	172	28	ffq 137	Pre-pregn	OGTT	1.01	0.65	1.57	SUN	Ham
23	Dong	Japan	2019	3	2	84,948	21.2	1904	30	ffq 172	Pre-pregn	OGTT	0.78	0.67	0.9	Japan Environm	chocolate
24	Hu	China	2019		2	1014		238		Three-Day	Midpregna	OGTT	0.73	0.46	1.16	Born in Shenyan	Sweet foods
25	Wen	China	2020		2	324		101		ffq	Midpregna	OGTT	1.97	0.94	4.12	Longitudinal Tw	Sweets-based pattern
26	Hehua	China	2021		2	2244	23	476	31.67	ffq	Pre-pregn	OGTT	0.86	0.59	1.25	Northeast Coho	Sweets-based pattern
27	Leone	Spain	2021	10.3	2	3730	21.4	186	26	ffq	Pre-pregn	OGTT	1.1	0.74	1.64	SUN	Ultra-Processed
28	Yisahak	USA	2022	4	2	1948	25.1	85	27.5	ffq	early	OGTT	0.99	0.46	2.11	prospective U.S.	Ultraprocessed
29	Yong	Malaysia	2021	2	2	452		48	30.01	ffq	early	OGTT	1.17	0.46	2.95	The Seremban C	Processed meat
30	Yong	Malaysia	2022	2	2	452		48	30.01	ffq	early	OGTT	0.97	0.93	1.02	The Seremban C	Chocolate drinks
31	Tsoi	China	2022	1	2	156	22.6	21	32.7	ffq	early	World Health C	1.16	0.58	2.31	Hong Kong Chin	Sweet and fast-food pattern
32	Zuccolotto	Brazil	2019	1	3	785		139	27.6	24-hour di	early	OGTT	0.96	0.59	1.55	Unified Health S	Snacks pattern
33	Mahendra	UK	2023		2	785				ffq 221	early	OGTT	0.72	0.5	0.89	BAngalore Nutri	fried and fast food
34	Sartorelli	Brazil	2019	1	3	785		139	28	24-hour di	early	OGTT	0.82	0.49	1.36	Unified Health S	ultra-processed food
35	Wahedy	Palestine	2021		1	210	28.8	70	27	ffq		World Health C	0.89	0.79	1.1		unhealthy
36	Lotfi-a	Iran	2020		1	341	27.03	173	30	24-hours food recall			3.3	1.2	9.1		sugares
37	Lotfi-b	Iran	2020		1	341	27.03	173	30	24-hours food recall			2.7	1.2	6.06		Fast food
38	Lotfi-c	Iran	2020		1	341	27.03	173	30	24-hours food recall			4.3	1.8	10.2		fried food
39	Sedaghat	Iran	2017		1	388	25.6	122	29.6	ffq	Pre-pregna	OGTT	1.68	1.04	2.72		western
40	Bartáková-a	Czechia	2018		1	363	22.5	293	33	ffq	Midpregna	OGTT	7.6	0.99	57.96		Creamy cakes, other cakes, chocolat
